# Prevalence of and reasons for women’s, family members’, and health professionals’ preferences for cesarean section in Iran: a mixed-methods systematic review

**DOI:** 10.1186/s12978-020-01047-x

**Published:** 2021-01-02

**Authors:** Mahboubeh Shirzad, Elham Shakibazadeh, Khadijeh Hajimiri, Ana Pilar Betran, Shayesteh Jahanfar, Meghan A. Bohren, Newton Opiyo, Qian Long, Carol Kingdon, Mercedes Colomar, Mehrandokht Abedini

**Affiliations:** 1 Department of Health Education and Promotion, School of Public Health, Tehran University of Medical Sciences, Second floor, Building Two, Poursina Avenue, Tehran, Iran; 2 Department of Health Education and Promotion, School of Public Health, Zanjan University of Medical Sciences, Zanjan, Iran; 3 Department of Reproductive Health and Research, UNDP/UNFPA, UNICEF/WHO/World Bank Special Programme of Research, Development and Research Training in Human Reproduction (HRP), World Health Organization, Geneva, Switzerland; 4 School of Public Health, Central Michigan University, Michigan, USA; 5 Gender and Women’s Health Unit, Centre for Health Equity, Melbourne School of Population and Global Health, University of Melbourne, Carlton, VIC Australia; 6 Global Health Research Center, Duke Kunshan University, Kunshan,, Jiangsu China; 7 School of Community Health and Midwifery, University of Central Lancashire, Preston, UK; 8 Montevideo Clinical Research Unit (UNICEM), Montevideo, Uruguay; 9 Maternal Health Department, Ministry of Health, Tehran, Iran; 10 Department of Public Health and Community Medicine, School of Medicine, Tufts University, Tufts, USA

**Keywords:** Cesarean section, Vaginal birth, Qualitative synthesis, Quantitative analysis, Mixed method, Iran

## Abstract

**Background:**

Cesarean section (CS) rates have been increasing globally. Iran has one of the highest CS rates in the world (47.9%). This review was conducted to assess the prevalence of and reasons for women’s, family members’, and health professionals’ preferences for CS in Iran.

**Methods and findings:**

In this mixed-methods systematic review, we searched MEDLINE/PubMed, Embase, CINAHL, POPLINE, PsycINFO, Global Health Library, Google scholar; as well as Iranian scientific databases including SID, and Magiran from 1 January 1990 to 8th October 2019. Primary quantitative, qualitative, and mixed-methods studies that had been conducted in Iran with Persian or English languages were included. Meta-analysis of quantitative studies was conducted by extracting data from 65 cross-sectional, longitudinal, and baseline measurements of interventional studies. For meta-synthesis, we used 26 qualitative studies with designs such as ethnography, phenomenology, case studies, and grounded theory. The Review Manager Version 5.3 and the Comprehensive Meta-Analysis (CMA) software were used for meta-analysis and meta-regression analysis. Results showed that 5.46% of nulliparous women (95% CI 5.38–5.50%; χ^2^ = 1117.39; df = 28 [p < 0.00001]; I^2^ = 97%) preferred a CS mode of delivery. Results of subgroup analysis based on the time of pregnancy showed that proportions of preference for CS reported by women were 5.94% (95% CI 5.86–5.99%) in early and middle pregnancy, and 3.81% (95% CI 3.74–3.83%), in late pregnancy. The heterogeneity was high in this review. Most women were pregnant, regardless of their parity; the risk level of participants were unknown, and some Persian publications were appraised as low in quality. A combined inductive and deductive approach was used to synthesis the qualitative data, and CERQual was used to assess confidence in the findings. Meta-synthesis generated 10 emerging themes and three final themes: ‘Women’s factors’, ‘Health professional factors’, andex ‘Health organization, facility, or system factors’.

**Conclusion:**

Despite low preference for CS among women, CS rates are still so high. This implies the role of factors beyond the individual will. We identified a multiple individual, health facility, and health system factors which affected the preference for CS in Iran. Numerous attempts were made in recent years to design, test and implement interventions to decrease unnecessary CS in Iran, such as mother-friendly hospitals, standard protocols for labor and birth, preparation classes for women, midwives, and gynaecologists, and workshops for specialists and midwives through the “health sector evolution policy”. Although these programs were effective, high rates of CS persist and more efforts are needed to optimize the use of CS.

## Introduction

The cesarean section (CS) rate has been increasing globally [1]. According to the latest data from 150 countries, currently, 18.6% of all births occur by CS, ranging from 1.4 to 56.4% [2]. Countries with the highest CS rates in each region are Brazil (55.6%) and Dominican Republic (56.4%) in Latin America and the Caribbean, Egypt (51.8%) in Africa, Iran and Turkey in Asia (47.9% and 47.5%, respectively), Italy (38.1%) in Europe, United States (32.8%) in Northern America, and New Zealand (33.4%) in Oceania[2].

The latest available figures suggest that this trend is continuing, while the global healthcare community has considered the optimal range for a caesarean section to be between 10 and 15% of all births [3], as rates higher than 10% are not associated with reductions in maternal and newborn mortality rates [4, 5].

This growing number of CS can lead to several problems for women, children (e.g. increased risk of asthma and obesity), and future pregnancies (e.g. increased risk of miscarriage and stillbirth) [6]. Moreover, CS creates significant challenges for healthcare systems [6, 7].CS has an economic burden and incremental costs for households and society [8].

In Iran, a six-fold increase in the CS rate has been reported; from less than 7% in the 1970s to over 48% in 2018 [3, 9–11]. The rate is even higher in private hospitals (72–89%) [12–15]. The causes of high CS rates are multifactorial; however, non-clinical indications for CS have become significant contributors to the increase[16]. Modifications in the characteristics of populations, such as an increase in the prevalence of obesity, increase in proportion of nulliparous women or older women have contributed to the rise [17, 18]. Other factors such as differences in clinicalpractice styles, increasing fear of medical litigation, as well as organizational, economic, social and cultural factors have all been implicated in this trend [19–22].

In 2014, the Ministry of Health and Medical Education (MoHME) in Iran conducted several structural and educational reforms to control the CS rise. In the structural reforms, vaginal deliveries became free of charge in all public hospitals; the physical infrastructure of labour wards was improved to increase women' privacy, and financial incentives were provided for the service providers for vaginal births (VBs) in public hospitals to encourage them to prevent unnecessary cesarean sections [23]. In educational reforms, the educational curriculums of midwifery students and obstetrics residents have been revised, and related guidelines [e.g. outpatient and inpatient obstetrics emergency guidelines) have been developed. Despite these policy actions, the CS rate remains high [24].

Several studies in Iran have explored the reasons behind the increasing CS rates. These studies have identified a range of factors including individual-level factors (fear of labour pain, perceived safety of CS, concerns about complications following vaginal delivery) [25], facility-level factors (inappropriatecommunication between medical staff and women) [26], and system-level factors (inadequate vaginal birth after cesarean section (VBAC) policy implementation) [11, 27].

Understanding the role of and reasons for women’s, family members’, and health professional’ preferences for mode of delivery in Iran can provide information to develop relevant policy and intervention strategies aiming to decrease unnecessary CSs. We conducted a mixed-methods systematic review to assess women’s, family members’, and health professionals’ preferences for mode of delivery in Iran to map the reasons for preferences for CS, including individual, health system, societal, and cultural factors worldwide. We expect the findings to provide evidence-based recommendations on non-clinical interventions for policymakers as well as for clinicians and other health professionals to reduce CS rates in Iran.

## Methods

This mixed-methods review is a part of a global review of women’s and health professionals’ preferences for CS. The protocol was registered in PROSPERO (registration number:CRD42016036596) [22]. This systematic review is reported in accordance with the Preferred Reporting Items for Systematic Reviews and Meta-Analyses (PRISMA) and Enhancing Transparency in Reporting the Synthesis of Qualitative Research (ENTREQ) [28] guidelines.

### Search strategy

We searched the following electronic databases for eligible studies from 1st January 1990 to 8th October 2019: MEDLINE/PubMed, Embase, CINAHL, POPLINE, PsycINFO, Global Health Library, Google scholar, and Iran databases including SID (Scientific Information Database), and Magiran. Search strategies were comprised of keywords and controlled vocabulary terms. The search strategy for each database is presented in Additional file 1.

In addition to the database searches, we also conducted ‘related article’ searches in PubMed for all studies included in the review. We also reviewed reference lists of include studies. We searched the reference lists of all the included studies and key references (i.e., relevant systematic reviews). We searched for any pertinent papers that might have cited the included papers and key references (i.e. forwards citation search) in the ISI Web of Science (both the Science Citation Index and Social Science Citation Index) and Google Scholar. All citations were imported into the EndNote, and duplicate studies were identified and deleted. Two review authors (Kh.H and M.Sh) screened the titles and abstracts of the identified records independently to evaluate potential eligibility; those that were irrelevant to the study topic were discarded. The full texts of all the potentially relevant papers were then retrieved and assessed based on the review’s inclusion criteria. At all stages, discrepancies and uncertainties were resolved by seeking a third review author’s (E.Sh) view.

### Inclusion and exclusion criteria

We included primary quantitative, qualitative, and mixed-methods studies conducted in Iran that investigated preferences of women and family members and health professionals for mode of delivery, and the reasons underlying such preferences.

In the quantitative component, we included studies that were cross-sectional, longitudinal studies, or baseline data from interventional studies. Inclusion criteria was (1) original research, (2) studies conducted in both urban and rural settings, (3) women’s views about their preferences for mode of birth during current pregnancy regardless of their obstetric characteristics (e.g. parity, pregnancy status and whether or not they have had a previous CS), or socio-economic status.

In the qualitative component, we included primary studies that used qualitative study designs (e.g. ethnography, phenomenology, case studies, grounded theory studies and qualitative process evaluations).We included studies that used both qualitative methods for data collection (e.g., focus group discussions, individual interviews, observation) and qualitative methods for data analysis (e.g. thematic analysis, framework analysis). We excluded studies that collected data using qualitative methods but did not analyze the data qualitatively (e.g., open-ended survey questions where the response data were analyzed using descriptive statistics only). We did not exclude any studies based on our assessment of methodological limitations but utilized this information to assess our confidence in the synthesis findings.

### Data extraction

**Fig. 1:**
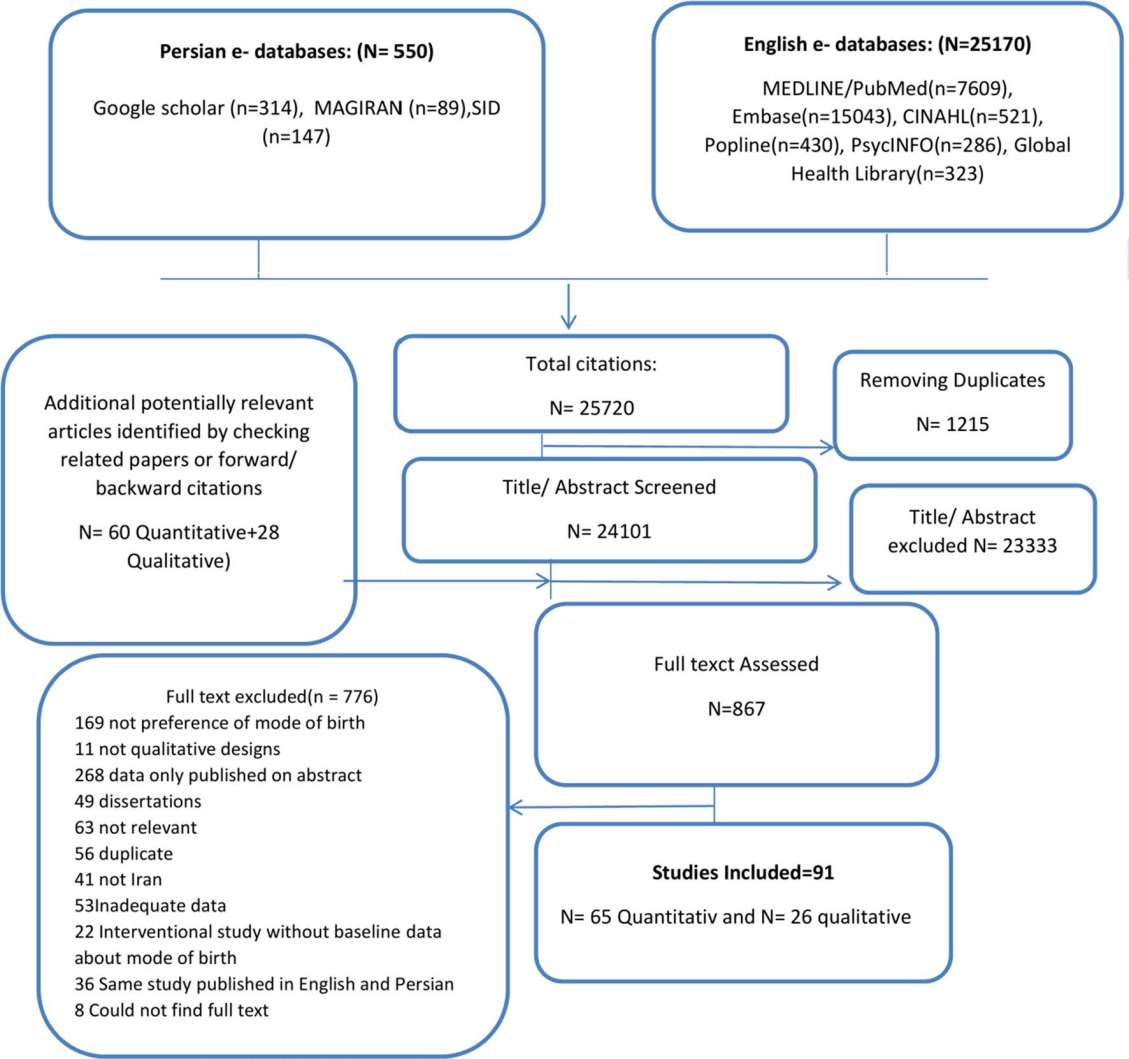
Flow chart of the study identification and selection

Data extraction was performed using a form designed specifically for this review (Additional file 2). Data were extracted by one review author (M.Sh) and checked by a second review author (Kh.H). Disagreements were discussed and resolved with a third review author (E.Sh). In the quantitative component, numerical data (frequency or percentages) were extracted related to preferences for mode of birth (Additional file 3). Study participants (nulliparous, multiparous), pregnant women with and without previous CS, residence (urban, rural, or both), and risk for pregnancy were extracted as covariates of the study. Funding sources were also extracted. For the qualitative studies, we extracted characteristics of the study, methods, and population; as well as the relevant themes, authors’ interpretations, and participants’ quotations (preference and reasons for mode of delivery). We contacted authors via email if the data in the original papers were not clear or if some details were missing. We included a flow diagram to show our search results and the process of screening and selecting studies for inclusion (Fig. 1).

### Assessment of the methodological limitations in included studies

In the quantitative component, two review authors (M.Sh and Kh. H) independently assessed methodological limitations for each research using a ten quality criteria tool developed by Long et al. [22] based on existing instruments for observational studies (STROBE, NEWCASTLE, and Circum Network’s Assessing Survey Research) [29–31]. The ten questions evaluated the reliability and quality of the information by assessing the eligibility criteria, sample size, representativeness, response rate, clarity of the questions/statements, ethical considerations, transparency of data (including numerators, denominators, and missing values), and consistency between the research question and data reported (Additional file 4). Each question had one score; hence each study could be given a score from 0 to 10. The total quality of quantitative research was classified based on the median score. If the score was lower than, the same as, or higher than the median score, the quality of the study was considered to be ‘low’, ‘middle’, or ‘high’, respectively. In the qualitative component, we used a checklist described by Walsh and Downe [32] for evaluating the quality of primary qualitative studies and the qualitative components from mixed-methods studies. Based on this checklist, studies were categorized in four degrees from A (few flaws) to D (significant flaws).We included studies that met our inclusion criteria regardless of study quality. The assessment of methodological limitations of both quantitative and qualitative studies is listed in the Additional file 5.

### Data analysis

We conducted the meta-analysis to analyse the proportions of preference for CS among the included primary quantitative studies using Review Manager Version 5.3 (RevMan; Cochrane Community, Oxford, UK). We also estimated the effect sizes and 95%CI using RevMan. We calculated the pooled proportion as the Freeman–Tukey variant of the arcsine square root of transformed proportion, using inverse variance weights for the random-effects model [33]. We conducted the subgroup analysis based on the parity (nulliparous or multiparous, if specified in the included studies), and the time when the preference was reported (early and middle pregnancy [first and second trimester], late pregnancy [third trimester], or gestational age not specified).

I^2^ statistic, a descriptive index that estimates the ratio of true heterogeneity, was used to quantify heterogeneity across the observed effect sizes. Significant heterogeneity was tested for (I^2^ > 40%). Whenever heterogeneity could not be explained by subgroup analysis and sensitivity analysis, we conducted meta-regression analysis using the Comprehensive Meta-Analysis (CMA) software, adjusting for study participants (nulliparous, multiparous), pregnant women with and without previous CS, residence (urban, rural, or both), and risk for pregnancy as defined by the study authors (low risk, high risk, and not specified). The CMA created forest and funnels plots and computed the rank correlation. We used the Q statistic to measure weighted squared deviations. A p-value for the Q-test below 0.1 indicated significant heterogeneity in the summary effect sizes. A common among-study variance across moderator subgroups was assumed when the 95% CI of effect size overlapped zero, and its p-value was less than 0.05. Funnel plots were used to aid visual identification of the presence of publication bias when more than ten studies were included. Funnel plots displayed the standard error for each study against the study's effect size. Reasons for preferring CS reported by the participant were mapped and grouped into several categories and were summarized as a brief narrative.

We used a combined inductive and deductive approach to thematic synthesise the qualitative data. Thematic synthesis methods were used to conduct initial open coding on each relevant text unit to elicit key themes emerging from the data [34, 35]. Thematic synthesis is recommended by the Cochrane Qualitative Review Methods Group [36].We also reviewed and considered existing resources to inform the organization of a preliminary thematic framework [37],which included the framework reported by Long et al. [22] and the WHO recommendations non-clinical interventions to reduce unnecessary cesarean sections [38] as a priori frameworks of themes and categories. Three review authors independently read and re-read the selected studies and applied the framework, moving between the data and the themes covered by the framework, but also searching for additional themes until all the studies had been reviewed and no new themes emerged. We then revised the framework in line with the ideas and categories that emerged from this synthesis.We later developed the thematic synthesis further by rearranging data according to the appropriate part of the thematic framework to which they related and formed charts. Our charts contained distilled summaries of evidence from different stakeholder perspectives and involved a high level of abstraction and synthesis.

### Assessment of confidence in the synthesis findings

Two review authors (E.Sh, Kh.H) independently used the GRADE-CERQual (Confidence in the Evidence from Reviews of Qualitative research) approach to summarise our confidence in each finding [39]. CERQual assesses confidence in the evidence, based on the following four key components:

- Methodological limitations of included studies: the extent to which there are concerns about the design or conduct of the primary studies that contributed evidence to an individual review finding [40].
- Coherence of the review finding: an assessment of how clear and compelling the fit is between the data from the primary studies and a review finding that synthesizes those data. By persuasive, we mean well supported or compelling [41].
- Adequacy of the data contributing to a review finding: an overall determination of the degree of richness and quantity of data supporting a review finding [39].
- The relevance of the included studies to the review question: the extent to which the body of evidence from the primary studies supporting a review finding applies to the context (perspective or population, the phenomenon of interest, setting) specified in the review question [40].

After assessing each of the four components, we made a judgment about the overall confidence in the evidence supporting the review finding. We judged confidence as high, moderate, low, or very low [42]. The final assessment was based on consensus among the review authors. All findings started as high confidence and were then graded down if there were important concerns regarding any of the CERQual components.

In keeping with quality standards for rigour in qualitative research, we considered our views and opinions (reflexivity) on mode of delivery as possible influences on the decisions made in the design and conduct of this review, including the search strategy, inclusion decisions, synthesis, and interpretation of the findings; and, in turn, on how the emerging results of the review influenced our views and opinions.

## Results

We identified a total of 65 quantitative [43–108] and 26 qualitative studies [27, 109–133] studies for inclusion in the analysis (Fig. 1). Table 1 shows the main characteristics of the included studies. Among the quantitative studies, most were conducted in urban areas; 29 studies (44.6%) involved nulliparous women; 35 studies (53.8%) involved pregnant women regardless parity, two studies (3.0%) involved health professionals (doctors and midwifes), one study (1.5%) included pregnant women’s family members, one study (1.5%) recruited pregnant women without previous CS, and one study (1.5%) included pregnant women with previous CS. Studies were supported by the related universities.

**Table 1:** Summary of characteristics of included studies

Characteristic	Number of studies	Studies
Total	91	[27, 45–135]
*Language of publication*		
Persian	55	[45, 55–89, 103–110, 114–120, 122, 123, 127, 129, 130]
English	36	[27, 46–54, 90–102, 111–113, 121, 124–126, 128, 131–135]
*Year of data collection*		
1999–2010	18	[47, 50, 54, 60–62, 65, 75–77, 79, 81, 83–87, 124]
2011 or 2019	56	[27, 45, 46, 48, 55–57, 64, 67, 72, 73, 78, 82, 90–95, 97–123, 125–135]
Not specified	17	[49, 51–53, 58, 59, 63, 66, 68–71, 74, 80, 88, 89, 96]
*Study design*		
Quantitative study	65	[45–110]
Longitudinal	1	[46]
Cross-sectional	48	[45, 47–54, 57–62, 65–69, 72–78, 80–84, 86–94, 96, 98, 100, 102, 103, 107–109]
Experiment (baseline)	15	[55, 56, 63, 64, 70, 71, 79, 85, 95, 97, 99, 101, 104–106]
Prospective cohort study	1	[110]
Qualitative study	26	[27, 111–135]
*Location*		
Urban	80	[27, 45, 47, 48, 50–56, 58–77, 79–89, 92–112, 114, 117, 118, 120–132, 134, 135]
Rural	1	[57]
Mixed	3	[78, 116, 119]
Unknown	7	[46, 49, 90, 91, 115, 113, 133]
*Population*		
Facility-based	89	[27, 45–47, 49–89, 91–135]
Population-based	1	[48]
Unknown	1	[90]
Participants		
*In quantitative study*		
Pregnant women (regardless parity)	33	[44–47, 52, 55, 56, 58–60, 62, 64, 65, 71–73, 75, 77–79, 82, 84, 85, 87, 91, 93, 97–102, 107]
Nulliparous	28	[43, 48–51, 53, 54, 57, 61, 63, 67–70, 74, 76, 83, 86, 89, 90, 92, 9, 96, 103–107]
Multiparous	3	[50, 90, 94]
Pregnant women with previous CS	2	[48, 88]
Mothers and husbands of women	1	[95]
Midwifes and doctors	3	[66, 81, 87]
*In qualitative study*		
Women (pregnant, postpartum, NVD or CS, Nulliparous or Multiparous), healthcare providers ( midwife, physicians), husbands	26	
*Quality of quantitative included studies*	65	
Low	18	[45, 46, 48, 51, 57–60, 64, 71–73, 81, 83–85, 89]
Middle	5	[50, 69, 70, 77, 80]
High	41	[47, 49, 52–56, 61–63, 65–68, 74–76, 78, 79, 82, 86–88, 90–110]
*Quality of qualitative included studies*	26	[27, 111–135]
A: High	2	[27, 113]
B: Moderate	12	[117, 118, 121, 124–126, 128, 130, 132–135]
C: Low	10	[111, 114–116, 119, 120, 123, 129, 127, 131]
D: very low	2	[112, 122]

Most of the qualitative studies were also been conducted in urban areas. Among the qualitative studies, seven studies (26.9%) involved health professionals (doctors, midwives, and healthcare providers), nine studies (34.6%) involved postpartum women, and 17 (65.4%) studies involved pregnant women.

### Quantitative results

#### Prevalence of women’s preference for CS

Sixty-five studies investigated the participant’s preference for CS[43–108]. First, we analyzed the data with RevMan, the heterogeneity was high (95% CI 145(145,146); χ^2^ = 3878.82; df = 62 [p < 0.00001]; I^2^ = 98%), hence the subgroup analysis as below.

While 5.46% of nulliparous women preferred a CS (95% CI 5.38%–5.50%; χ^2^ = 1117.39; df = 28 [p < 0.00001]; I^2^ = 97%) [43, 48–51, 53, 54, 57, 61, 63, 67–70, 74, 76, 83, 86, 89–96, 103, 105, 106, 108], this proportion was 53.05% for multiparous women (95% CI 51.66%–51.44%; χ^2^ = 144.70; df = 2 [p < 0.00001]; I^2^ = 99%) [48, 88, 92]. However, 35 studies did not specify whether the participants were nullipara or multipara, and the proportion of preference for CS in this group of women was 2.06% (95% CI; 2.05–2.08%; χ2 = 2133.04; df = 34 [p < 0.00001]; I^2^ = 98%) [44–48, 52, 55, 56, 58–60, 62, 64, 65, 71–75, 77–79, 82, 84, 85, 87, 93, 97–102, 107].

The results of subgroup analysis based on the timing during pregnancy showed the proportions of preference for CS reported by women in the early and middle pregnancy, late pregnancy, and gestational age not specified. For women in early and mid-pregnancy, 5.94% preferred CS (95% CI 5.86–5.99%; χ^2^ = 194.59; df = 11 [p < 0.00001]; I^2^ = 94%) [46, 49, 50, 55, 61, 68–70, 72, 85, 102–104]. Among women who were in the third trimester (late pregnancy), this proportion was 3.81% (95% CI 3.74%–3.83%; χ^2^ = 549.67; df = 23[p < 0.00001]; I^2^ = 96%) [43, 44, 46, 48, 49, 51, 53–57, 62, 64, 72, 76, 77, 83, 90, 92, 93, 95, 96, 105]. Preference for CS in studies that did not specify gestational age of women was 3.7% (95% CI 3.76–3.81%; χ^2^ = 2865.90; df = 31 [p < 0.00001]; I^2^ = 99%) [45, 47, 52, 58–60, 63, 65, 67, 71, 73–75, 78, 79, 82, 84, 86–89, 94, 97–101, 106–108].

**Fig. 2:**
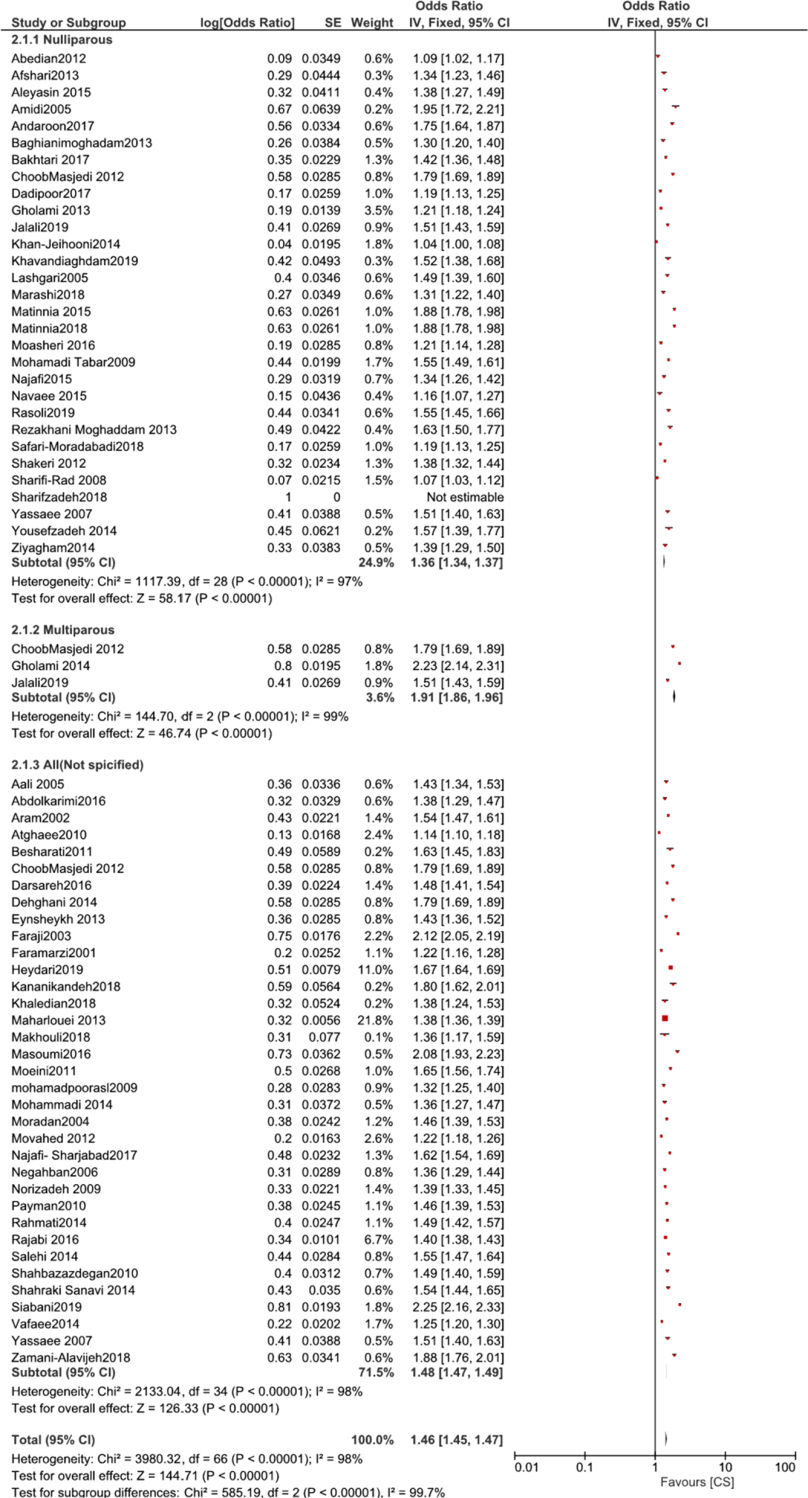
Forest plot of comparison: proportion of CS preference based on parity

**Fig. 3:**
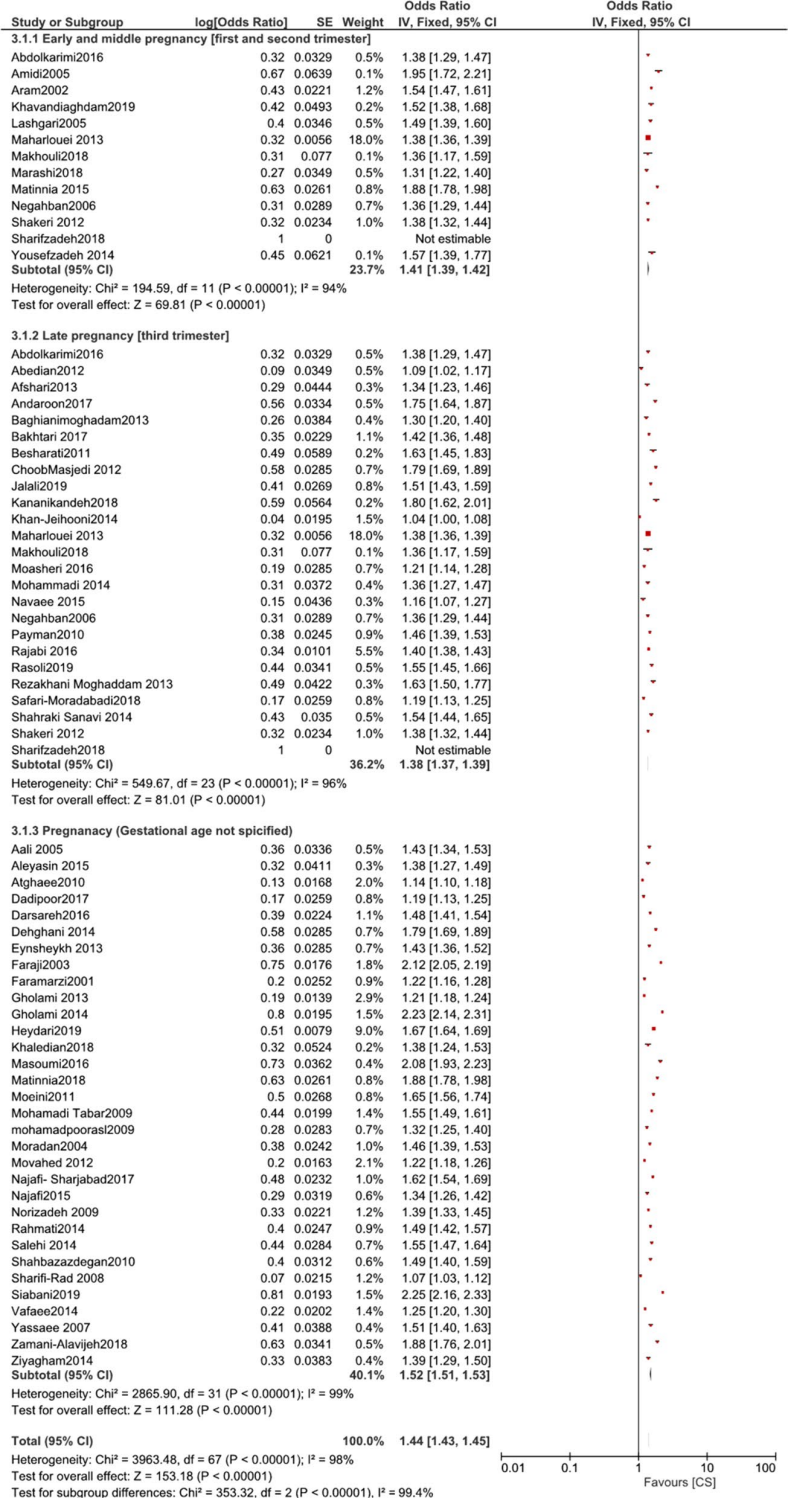
Forest plot of comparison: proportion of CS preference based on time of pregnancy

Figures 2 and 3 shows forest plots of the proportions of women preferring CS based on parity (nulliparous or multiparous, if specified in the studies), and time when the preference was reported ([first and second trimesters] or [third trimester], or gestational age not specified).

**Fig. 4:**
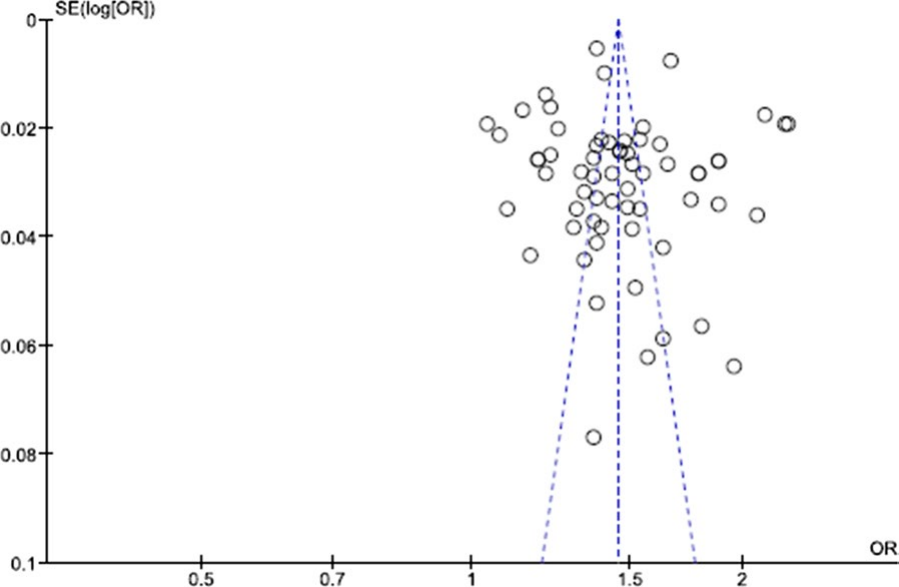
Funnel plot of comparison: proportion of CS preference, outcome: CS preference

The results showed that heterogeneity was higher than I^2^ > 40%. The heterogeneity could not be explained by subgroup analysis; hence we conducted the meta-regression analysis. Figure 4 shows the funnels plots of the proportion of participants preferring CS. Publication Bias did not affect the results obtained, as shown by the presence of symmetry in the funnel plot. Egger's test results also confirm the results.

The outcome variable (event rate of CS) is predicted according to the values of four explanatory variables (women, timing during pregnancy, type of study, and risk for the pregnancy). The result of study showed just three explanatory variables (women, risk for the pregnancy, and type of study) could predict the event rate of CS. The regression coefficient obtained from a meta-regression analysis describes how the outcome variable (event rate of CS) changes with one unit increase in the explanatory variable (Additional file 6).The R2, Test of model, and Goodness of fit were also compared based on each model. The comparison of the models is shown in Additional file 7.

The regression coefficient gives an estimate of the relative change in effect size with a unit increase in the explanatory variable. Based on the results of this study, the regression coefficient (R2) indicates that 31% of the variation of the dependent variable (event rate of CS) is explained by the independent variables (women, risk for the pregnancy, and type of study).

*Reasons for preferring CS* Fourteen quantitative studies reported reasons for women’s preference for CS (Additional file 8) [49, 56–58, 60, 72, 81, 82, 85, 87, 95, 97, 99, 107], which were summarized into eleven categories (Table 2). Across studies, the most common reasons underlying the preference for CS were pain-related fear of VB (with the proportion of women giving this reason ranging from 37.2 to 77%) [49, 56–58, 60, 72, 81, 82, 85, 97, 99, 107], fear of vaginal damages (8.8 to 64.67%) [72, 81, 87, 97, 99], and the perceived risks of vaginal delivery for the baby (e.g., fear of risk for baby (6.2 to 75.33%) [49, 56, 57, 72, 81, 85, 87, 99]. Other causes are outlined in Table 2.

**Table 2:** Reasons for preference for caesarean section reported by women, quantitative studies

	Preference for current pregnancy or index birth														
Study	Payman-2010	Andaroon-2017	Moradan-2004	Norizadeh-2009	Bani-2012	Bani-2012	Negahban-2006	Shahbazazdegan-2010	Aram-2002	Vafaee-2013	Shakeri_2012	Rasoli-2019	Najafi- Sharjabad2017	Siabani-2019	Masoumi-2016
Location	Mashhad	Mashhad	Semnan	Marand	Tabriz	Tabriz	Rafsanjan	Ardebil	Esfahan	Shiraz	Zanjan	Mazandaran	Bushehr	Kermanshah	Hamedan
Study population	Pregnant women	Nulliparous	Pregnant women	Pregnant women	Doctors	Midwives	Pregnant women	Pregnant women	Pregnant women	Male partners	Nulliparous	Nulliparous pregnant women	Pregnant women	Pregnant women	Pregnant women
Sample size	390	220	400	450	153	90	256	245	500	417	397	211	462	410	150
Reasons for preference															
Pain-related fear															
Fear of labour pain	59%	47.2%	59%	47.6%	47.3%	77%	52.2%	**43.3**%	37.2%	NR	43.3%	NR	39.5	61.46	65.33
Fear of NVD -perceived maternal short-term risks															
CS is safe and reliable	NR	NR	NR	NR	34.5%	NR	NR	NR	NR	NR	NR	NR	NR	NR	NR
Safer for mother by CS	NR	NR	NR	NR	NR	NR	3.8%	NR	NR	NR	NR	NR	NR	NR	NR
To reduce the damage of the pelvic floor	NR	NR	NR	2%	NR	NR	NR	NR	NR	NR	NR	NR	NR	41.22	NR
Fear of NVD—perceived maternal long-term risks															
Better keeping body image by CS	2.7%	NR	NR	NR	NR	NR	NR	NR	6.5%	NR	NR	NR	NR	NR	NR
Fear of vaginal damages	NR	NR	NR	NR	NR	57.5%	8.8%	NR	NR	34.8%	NR	NR	NR	58.78	64.67
Fear of urinary incontinence	NR	NR	NR	NR	NR	57.6%	NR	NR	NR	NR	NR	NR	NR	58.78	NR
Maternal health	NR	4.1%	NR	NR	NR	NR	NR	NR	NR	NR	NR	NR	NR	NR	NR
Fear of VD -perceived risks for the baby															
Fear of risk for baby	12.8%	19.5%	NR	NR	70.9%	NR	6.2%	NR	11.5%	29.3%	**33.4**%	NR	NR	NR	75.33
Perceived that baby would be more clever	NR	NR	NR	0.2%	NR	NR	NR	NR	NR	NR	NR	NR	NR	55.61	NR
Birth trauma to the new-born	NR	NR	NR	NR	67.2%	71.4%	NR	NR	NR	NR	NR	NR	NR	40.97	NR
Cultural and societal related beliefs															
- Reasonable for schedule and able to select “lucky date” for the birth	NR	NR	NR	2.6%	NR	NR	NR	NR	NR	NR	NR	NR	NR	NR	NR
-Do not like the position of NVD	NR	NR	NR	NR	NR	NR	NR	NR	NR	NR	NR	NR	NR	55.61	NR
Women's experience															
Prior CS	NR	NR	NR	18.2%	NR	NR	NR	19.1%	NR	NR	NR	NR	28.5	NR	NR
PriorAbortion / infertility	NR	NR	5.3%	NR	NR	NR	NR	NR	NR	NR	NR	NR	NR	NR	NR
Prior negative experience from NVD	6%	NR	3.9%	NR	NR	NR	NR	NR	NR	NR	NR	NR	NR	NR	NR
Health system factors															
Fear of environment	2.7%	NR	NR	NR	NR	NR	NR	NR	NR	NR	NR	NR	NR	NR	NR
Mistrust to stuff	NR	12.2%	NR	NR	NR	NR	NR	NR	NR	NR	NR	NR	NR	NR	NR
Significant others	2.7%	NR	NR	NR	NR	NR	NR	NR	NR	NR	NR	NR	NR	NR	NR
Bad story about NVD (by family, friend,..)	9.4%	17.1%	NR	NR	NR	NR	NR	NR	NR	NR	NR	NR	NR	NR	NR
doctors/midwives advice	36.2%	NR	NR	18.7%	NR	NR	NR	17.4%	35.8%	NR	NR	NR	32	NR	NR
Spouse/relative advice	NR	NR	NR	2/3%	NR	NR	5%	NR	4%	NR	NR	39.3%	NR	NR	NR
Tube ligation	NR	NR	7%	6.7%	NR	NR	17.5%	NR	NR	NR	1.6%	NR	NR	41.95	NR
Anxiety and sychological pressures	NR	NR	NR	NR	NR	NR	NR	NR	NR	NR	14.7%	NR	NR	NR	NR
Unknown factors	NR	NR	18%	NR	NR	NR	NR	NR	NR	NR	7%	NR	NR	NR	NR

### Qualitative synthesis

Of 26 qualitative studies (Additional file 9), 20 studies included the views of women [27, 109, 110, 112–116, 118–120, 122, 123, 125–129, 131, 133]; and seven studies explored the views of health professionals [111, 117, 121, 122, 124, 130, 131]. The earliest included study was published in 2009 [121, 123], the most recent in 2016 [109].

*Description of themes* Meta-synthesis generated ten emerging themes and three final themes: ‘Women’s factors’, ‘Health professional factors’, and ‘Health organization, facility, or system factors’. Table 3 presents the summary of qualitative review findings and CERQual assessments. Additional file 10 shows the CERQual evidence profiles of the review findings. Additional file 11 summarises initial concepts, emergent themes, final themes, and supporting quotes. Key results across themes are presented below.

**Table 3:** Summary of qualitative review findings

Summary of review findings	Studies contributing to the review finding	CERQual assessment of confidence in the evidence	Explanation of CERQual assessment
Theme 1:Women’s and health professionals’ beliefs			
*Deep rooted fear of labour pain and vaginal birth:* “Fear” was reported frequently by most of the women as one of the most important influencing factor on choosing mode of delivery; and fear from pain was the most common cause of fear	[109, 110–120, 122–125, 127, 129, 131, 132]	Moderate confidence	Due to minor concerns about methodological limitation and coherence
*Irreversible damage to body and sexual function*: Women believed that vaginal delivery would damage their genitalia and caused vaginal relaxation that led them to undergo genital cosmetic/medical surgeries. They believed that CS was an ideal method to maintain their figure and sexual satisfaction. Women believed that these kinds of damages would hurt their sexual function	[109–111, 113, 114, 120–123, 125, 129, 131–133]	Moderate confidence	Due to minor concerns about methodological limitations; No or very minor concerns about coherence and adequacy; and moderate concern about relevance
*Safety (mother/ baby) and comfort:* Many women believed that safety of the baby was guaranteed during CS; and CS is less traumatic for baby. Some women believed that children born by CS are more intelligent. The safety issues were more prominent if the baby was boy	[109–111, 113, 114, 116, 118, 119, 121–123, 125–127, 129, 130, 132]	Moderate confidence	Due to moderate concern about methodological limitations and minor concern about coherence
*Social convenience of birthing to time (time scheduling):* Some women preferred CS because they preferred to know the exact time of delivery. Some obstetricians also believed that women prefer to have a scheduled delivery	[109, 110, 112, 113, 117, 119, 120, 123, 125, 128]	Low confidence	Due to minor concerns about methodological limitations, and moderate concerns about relevance and adequacy
*Religious beliefs:* Although most women stated that vaginal delivery had severe pain, some indicated advantages of tolerating pain during childbirth that was a reflection of religious beliefs. Some women believed that vaginal delivery was a natural way of childbearing and considered it as God’s will. Also they believed that vaginal delivery was part of being a mother	[109, 112, 118–122, 128, 130]	Moderate confidence	Due to moderate concerns about methodological limitations; no or very minor concerns about relevance, coherence, and adequacy
*Cultural beliefs (having role models; modernity, capability to do vaginal birth):* CS was considered to be a higher class method of birth that people with a higher socio-cultural class and higher education preferred to choose it and it was a social norm. This cultural belief was stated by doctors as well. Having role models also play important role in women’s decision-making. Wealthy women or doctors and midwives behaviors were important	[111, 119, 121, 122, 124, 125, 128, 130, 131]	Moderate confidence	Due to minor concerns about methodological limitations and relevance
*Influence of information about birth from family, friends, doctors, and media:* Women, especially nulliparous ones, were eager to hear about the experience of their relatives and friends about different types of delivery. Some women mentioned that their fear was caused by negative experience of relatives and friends with regard to vaginal delivery. Some women reported recommendations from their mothers or husbands to undergo CS. Healthcare providers believed that non-standard birth facilities make unpleasant experience to women and they transfer these negative experiences to other women. Some women also reported stories of relatives or friends who had experienced inappropriate, unfriendly or even impolite behavior of labour and delivery ward staff. Some participants explained that their clinicians had a significant role in decision-making for choosing CS	[109–112, 114–116, 117, 119–129, 131, 132]	Moderate confidence	Due to moderate concerns about methodological limitations and relevance; minor concerns about coherence
*Women’s previous birth experience:* Previous undesirable experience had caused some women decide to have CS. Some women, who had been hospitalized during pregnancy due to complications such as hypertension, stated that the ward’s atmosphere (practicing students, lack of privacy, frequent vaginal examinations, etc.) frightened them and made them to choose CS for delivery. Some women reported that watching movies in which women were in pain due to vaginal delivery provide them a bad experience and they have decided to undergo CS. However, some women with previous childbirth were more likely to be in favor of vaginal delivery	[109, 112, 115, 118, 123, 124, 126, 127, 131]	Moderate confidence	Due to minor concerns about methodological limitations and moderate concerns about coherence
*Women’s preferences informed by availability (i.e. what they or insurance can pay): *Supplemental insurance plans in private hospitals support elective CS by providing high-quality facilities for women, Women who were not covered by these supplemental insurance plans could not pay for CS had not received insurance and they “had to” go vaginal delivery. Some women stated that after the “Health revolution program” and freeing vaginal deliveries, families were more eager to have vaginal delivery	[112, 119]	Moderate confidence	Due to minor concerns about methodological limitations and relevance; and moderate concerns about adequacy
Theme2: Healthcare professional factors			
*CS is now safe/r option for birth:* Some obstetricians deeply believed that CS was the better choice for both women and their babies. Unpredictable status of vaginal delivery and safety of baby were frequently stated reasons by doctors	[111, 117, 121, 124, 131]	Moderate confidence	Due to minor concerns about methodological limitation and adequacy
*Convenience of birthing to time (work scheduling):* Some doctors stated that the process of vaginal delivery is time consuming and unpredictable and disturbs night sleeps. They believed that they are too busy to pay time for vaginal delivery	[109, 111, 114, 117]	Moderate confidence	Due to moderate concern about methodological limitation and minor concern about adequacy
*Patient pushes doctor to do CS:* Doctors believed that reduced number of pregnancies as well as the increased age of marriage and pregnancy was leading to the families’ higher tendencies towards undergoing CS. Some doctors stated that one of the factors affecting the rise in CS is that the women and their families asked for a CS and pushed the doctor to do CS	[111, 117, 124]	Moderate confidence	Due to minor concern about methodological limitation and moderate concern about adequacy
*Legal issues:* Some of the proclamations made by the doctors showed the importance of legal issues in increasing rate of CS. Doctors stated that there were no guidelines or scientific basis which would guarantee the judging process. They believed that the policies and laws affect the behavior of healthcare providers	[111, 117, 121, 132]	Moderate confidence	Due to moderate concerns about methodological limitation and adequacy
*Vaginal delivery fees not worth the time paid for it:* A financial incentive in terms of higher fees for doctors in doing CS in private hospitals was considered to be a factor increasing the CS rate. Some Obstetricians also claimed that the fee paid for vaginal delivery is not worth the time consumed and stress endured during such a procedure. Changing the tariff imposed on vaginal delivery may be one of the strategies adopted by the policymakers to reduce CS rate	[111, 117, 121, 124, 132]	Low confidence	Due to minor concerns about methodological concern and coherence; moderate concerns about adequacy and no or minor concern about relevance
*Lack of respectful, dignified, and supportive communication with women:* Women stated that disrespect, poor communication between them, their families and healthcare providers and mistreatment could result in deciding not to go for vaginal delivery. Some women had bad experiences about mistreatment in labor that inhibited them from going back to labour ward for the next delivery	[27, 111, 112, 114, 115, 118–120, 123–125, 127, 128, 131, 132]	Moderate confidence	Due to moderate concerns about methodological limitation and minor concerns about coherence
*Lack of adequate information support:* Maternal unawareness regarding labor along with women’s imprecise knowledge about different delivery methods, their complications, and their hospitalization period has reduced their tendency toward undergoing a vaginal delivery. Both women and healthcare providers believed that providing maternity preparation classes and hotlines could help women to make proper decisions and made them ready for a vaginal delivery and reduce their stress	[27, 110, 111, 112, 114, 121, 122]	Moderate confidence	Due to moderate concerns about methodological limitation
*Mistrust:* Some women described the level of trust in their doctor as a factor in choosing their method of childbirth. Some stated that they did not trust the recommendations made by their doctors. Some doctors also stated that the patients did not trust them; and in case of complications, patients saw it as doctors’ fault,	[27, 111, 119, 124, 125, 132]	Moderate confidence	Due to minor concern about methodological limitation and relevance; and moderate concern about adequacy
*Lack of skilled and experienced doctors/midwives during labor and vaginal birth:* Many healthcare providers believed that the skills and experience of obstetricians and residents in conducting a vaginal delivery has been reduced in recent years due to poor quality of education. They believed that because of reduced number of birth rate in recent years, residents had rare opportunities to do vaginal deliveries	[27, 111, 114]	Moderate confidence	Due to minor concern about methodological limitation; moderate concerns about adequacy; no or minor concerns about coherence and relevance
Theme3: Health organization, facility, or system factors			
*Physical condition of birth facility (comfortable, calming, clean birth environment):* Poor quality care for women and their children during labor was the most commonly cited external barrier for vaginal delivery. Low environmental facilities, lack of proper equipment, and crowdedness were cited as low quality physical condition of birth facilities. Doctors and midwives also believed that the physical environment of labor rooms was far from standard. This unsuitable condition would negatively affect the women’s perspective and subsequently her decision regarding type of delivery	[27, 109, 111, 114, 115, 118, 119, 120, 121, 124125, 129, 131]	Moderate confidence	Due to moderate concerns about methodological limitation
*Physical examination and procedures (asking permission, privacy, painful vaginal examination, unnecessary vaginal examinations/interventions):* Some healthcare providers considered the early admission of women as a reason of unnecessary interventions, and consequently CS. Some of the midwives added that induction in patients with no evidence-based indication may also increase the C-section rate. Most doctors claimed that medicalizing the process of labor and adding interventions (such as hospitalizing, maintaining an IV-line and injecting solutions, elective induction and frequent vaginal examination) are among the factors turning physiologic labor into a non-physiologic process and consequently increasing the CS rate	[27, 109, 110, 111, 118, 119121, 123, 125, 129, 131]	Moderate confidence	Due to moderate concerns about methodological limitation
*Continuous, organized, timely care:* Fear of being alone during birth encompassed feelings of loneliness, being ignored by care providers, and feelings of helplessness were common fears expressed by women. Doctors also believed that the absence of an on call physician as an obstacle in the way of performing vaginal. Having a continuous midwifery care was proposed by some midwives	[27, 111, 114, 121–124]	High confidence	
*Limited availability of pain relief procedures:* Both doctors and women believed that providing a comfortable condition might hasten the tendency of vaginal delivery	[27, 112, 114, 118, 120, 124, 129]	Moderate confidence	Due to moderate concern about methodological limitations
*Lack of partner/family companion during labour/delivery:* Midwives or other healthcare providers are the women's only source of support during labour and childbirth because pregnant women are not allowed to have family companion during labor and birth in Iran. Having companions in labor that accompanied women during birth were mentioned by both women and healthcare providers as a supportive factor for parturient women	[27, 111, 120, 124, 128, 132]	Moderate confidence	Due to minor concern about methodological limitation and relevance
*Lack of practical birth guidelines and collaborative midwife-obstetrician models of care:* The absence of a scientific and accurate hospital protocol has also contributed to the addition of unnecessary and often non-scientific interventions to the labor process	[27, 111]	Very low confidence	Due to serious concerns about adequacy
*Too little value placed on midwifery care:* There have been changes in professional roles of midwives and obstetricians during childbirth. Midwives, who used to manage normal delivery and play a critical role in promoting physiologic labor, have lost their authority; and have faced challenges in realizing their role during birth. Midwives, who used to provide prenatal care at public healthcare centers, can no longer be actively involved in child delivery. Midwives and midwifery students account for less active involvement in vaginal delivery and subsequently a decline in the quality of their education has been occurred. Moreover, setting tariffs for labor affects the relation between physicians and midwives	[27, 111, 119, 124]	Moderate confidence	Due to minor concerns about methodological limitation and adequacy
*Financial and legal conflicts:* Many midwives claim that physicians receive all the money so why should a midwife spend long hours in the labor room; physicians, on the other hand, claim they should receive more money as they are in charge of any possible legal problems linked to labor	[111, 121]	Very low confidence	Due to serious concern about adequacy

### Women’s factors

#### Women’s and health professionals’ beliefs

##### Deep-rooted fear of labour pain and vaginal birth

“Fear” was frequently reported by most of the women as one of the most important influencing factors on choosing a mode of birth, and fear from pain was the most common cause of fear [109, 110, 112–120, 122–125, 127, 129, 131, 132]. Women felt that vaginal delivery was equivalent to pain and CS was equal to painlessness. A woman reported that she had postponed her pregnancy for five years because of fear from vaginal delivery pain: “While I am afraid of an injection, how can I do vaginal delivery” [123]. The extent of pain was described by women with suggestions like fear of death from excessive pain [122].

“Fear of mysterious” was also stated by some women [123, 125]; women did not like the unpredictable nature of vaginal delivery [119]. Some women had controversial feelings about birth pain. They felt it was simultaneously scary, good, and lovely [123]. Women with these feelings stated that they knew it was painful, but felt that they had self-control to cope with it [116], and believed that they had to experience pain only for a short period [109], and they would forget the pain afterwards [114].

Mostdoctors and midwives also believed that fear of labour pain had increased women’s preferences toward C-section. They said one of the ways to tackle the concern was to teach women about the the real nature of these pains [111].

##### Irreversible damage to body and sexual function

Women believed that vaginal delivery would damage their genitalia and caused vaginal relaxation that led them to undergo genital cosmetic/medical surgeriesin the future [109, 113, 114, 120, 122, 123, 125, 129, 131–133]. They believed that CS was an ideal method to maintain their figure and sexual satisfaction: “I think the womb will lose its original form. Thus, I do not like to have a normal delivery. Yes, it is good to have a normal delivery, but I do like to keep my shape” [109, 132]. One woman in the postnatal period stated: “Costs did not matter to me, because I did not need to do genital repair” [122]. Women believed that these kinds of damages would hurt their sexual function [122, 131]. Some women stated that vaginal delivery raised the likelihood of episiotomy local infections [129] and delayed initiating sexual activity [121].

Many women stated that their husbands asked them to undergo CS due to their husbands’ concerns about sexual function [120–122, 129] and they were ready to pay more money on it: “My husband said if in this hospital they don't perform CS, I'm prepared to spend a few million Tomans [the Iranian currency] to do CS in another hospital. He also said, ‘Even if I am forced to borrow money, I will not let you do VB’ [123].Some doctors also stated that women and their husbands are aware of genital complications of vaginal birth (pelvic relaxation) and its effect on sexual relationships.This awareness, along with the fact that Islamic law does not protect women with such disabilities (religious laws allow men to remarry and have multiple wives) has made families have a higher tendency toward CS to avoid this risk[111].

Some women believed that CS causedovarian cysts over time or chronic backpain [113].

##### Safety (mother/ baby) and comfort

Many women believed that the security ofthe baby was guaranteed during CS; and CS was less traumatic for baby [109–111, 113, 114, 116, 118, 119, 121–123, 125–127, 129, 130, 132]: “I knew it guarantees the health of my baby” [130]. Women started their fear of fetal birth injuries through vaginal delivery [109, 123, 132]: “It's better for the baby, for having a cesarean; my baby is getting compressed along the birth canal resulting it to be lack of oxygen” [132]. Some women believed that children born by CS are more intelligent [127]. The safety issues were more prominent if the baby was a boy. A midwife said that if the baby were boy, parents would ask the doctor to do CS [121].

Some women believed that their husbands preferredvaginal delivery; they indicated that they thought vaginal birth was a safer method for the mother and her baby [109].

On the other hand, some studies showed that concerns regarding baby’s health made women more in favour of vaginal delivery: “I think the most enjoyable moment for each mother is the moment that the baby is born naturally … you can hear its cry and be sure about its health” [126]. Some thought that anaesthesia has adverse effects on mother and baby’s health [113]. Women believed that vaginal delivery causes better feeding to babies and the success of women in breastfeeding. It also created better relationship between mother and baby [114]. In one study, some pregnant women stated that through vaginal delivery, toxins were eliminated from body and body regained its health [130].

##### Social convenience of birth time (time scheduling)

Some women preferred CS because they preferred to know the exact time of delivery [109, 110, 113, 117, 119, 120]: “I can do my works …in vaginal birth, a baby may come every moment, … at midnight, …, doctor maybe not accessible” [120].

Some women reported that CS is a natural, comfortable, and quick way of childbearing, and they need not experience any further stress. They disliked the idea of giving birth vaginally because it could be a time-consuming procedure [110, 112, 119, 123, 125, 128]: “I will go to the hospital at a specific time, I will be unconscious, and doctors would deliver my baby. Then, I will stay at the hospital for a night and come back home the day after. But when I think about vaginal delivery, I get scared” [119].

In one study, some doctors also believed that women prefer to have a scheduled delivery so that the women would know the exact time of delivery [117].

### Religious beliefs

Although most women stated that vaginal delivery had severe pain, some indicated advantages of tolerating pain during childbirth that was a reflection of religious beliefs [109, 112, 118–122, 128]: "Praying and seeking help from God and the saints give me power and enable me to endure labour pain" [122].

Some women believed that vaginal delivery was a natural way of childbearing and considered it as God’s will: “My preference for normal delivery is that I believe God had some good reasons for vaginal delivery … It seems that there should be some positive hidden reasons for the mother and baby in natural delivery. When a woman tolerates pain in natural delivery, her sin will be forgiven” [109] and “a symbol of God’s power, a divine gift which is not endowed to everyone, and a means of finding God” [130]. Also, they believed that vaginal delivery was part of being a mother-motherhood only could be achieved if they experienced vaginal delivery [109, 119].

A woman stated that she believed that tolerating birth pain will cleanse her of sins,, but she thought that the experience of pain is beyond imagination [120, 130]. They felt that if the expecting mother died while giving birth, she could reach the sublime degree of martyrdom [130].

Women suggested some strategies to cope with birth pain and boost psychological and spiritual strength during delivery, such as praying, praising God, promising offerings to God, and recourse to ‘Ahlulbayt’ were a few of them and reading Qoran [130].

### Cultural beliefs (having role models; modernity, capability to do vaginal birth)

CS is associated with prestige for many women. This belief plays a vital role in women’s decision-making process. CS was considered to be a higher class method of birth [111, 119, 121, 122, 124, 125, 128, 130] that people with a more upper socio-cultural class and higher education preferred to choose it and it was a social norm: “… and because of the high rate of performing cesarean surgery, it is better to do CS now” [128]. This cultural belief was stated by doctors as well [111, 124, 130]. They believed that people put more value on CS and appreciate doctors more if they had a CS [124]. They stated that it was a blind imitation by women [130].

They have role models that also play an essential role in women’s decision-making. Wealthy women or doctors and midwivesbehaviourswere important: “I have heard that none of the doctors use vaginal delivery, so cesarean is good” [130, 131]. A doctor stated that nowadays, most of the doctors undergo CS, and they are women’s role models: “When my colleague has undergone CS for a three-kilogram baby, how she could recommend vaginal deliveryto her patients?” [124].

Culturally, paying the cost of CS reflected the love and interest of husband to wife and also his concern in providing comfort to her, as expressed by women: “My husband said: ‘Do cesarean section, I will pay all its costs, I cannot see your pain during VB” [122]. Some women believed that if they did not go for CS, people might think they could not afford CS, and this was a cultural stigma for them [124, 130]: “If you spend money, doctors take care of you much better. So, I want to have a c-section because it is more expensive” [130].

### Women’s experiences

#### Influence of information about birth from family, friends, doctors, and media

Some women received information and stories regarding the mode of delivery from family and friends [109–112, 114–116, 119–128, 131, 132]. Women, especially nulliparous women, were eager to hear about the experience of their relatives and friends about different types of delivery. Some women mentioned that their fear was caused by negative experience of relatives and friends concerning vaginal delivery: “In my case, my colleagues’ views were beneficial to me because some of them who delivered in cesarean method said the delivery had no pain and ensured baby’s health” [131] or “My sister had a prolonged vaginal delivery with a lot of stitches, and it was very traumatic ….after one year she found a prolapsed vagina and some trouble in sexual activity. She had to have surgery, and her second pregnancy must be CS. She always recommended CS to all pregnant women because of some problems after vaginal delivery” [132].

Some women reported recommendations from their mothers [119, 121–123, 125, 132] or husbands [110, 111, 114, 119, 122, 129, 131]to undergo CS: “My mother herself has already uterine prolapse. So she always emphasizes me to have cesarean” [123].

Healthcare providers believed that non-standard birth facilities make an unpleasant experience for women, and they transfer these negative experiences to other women [114, 127]. Some women also reported stories of relatives or friends who had experienced inappropriate, unfriendly or even impolite behaviour of labour and delivery ward staff [127, 129, 132].

Some participants explained that their clinicians had a significant role in decision-making for choosing CS [112, 119, 122, 128]. Some women believed that midwives tried to convince them to have a normal delivery and midwives’ pieces of advice were one of the influential factors for choosing the delivery method [126]. Some women reported that their doctors recommended them to have CS and assured them about its safety [109, 110, 117, 119, 120, 122, 132]: “…most doctors are in favour of cesarean section” [109]. One participant reported that her doctor had said that ‘if I have a planned CS, she will guarantee to make the best surgical team, but if she needs emergency CS, she will not. My doctor said that if I wanted an elective section, I could have one. It's my right, and it is legal” [132].

Some women reported that watching movies in which women were in pain due to vaginal delivery provides them with a bad experience, and they have decided to undergo CS [124, 129].

#### Women’s previous birth experience

Previous undesirable experiences had caused some women to decide to have CS [112, 115, 118, 123, 126, 131]: “I have had a vaginal birth and bad memories from my previous delivery. I’m afraid of vaginal childbirth again, but I think it’s better than Caesarean section” [126]. Some women, who had been hospitalized during pregnancy due to complications such as hypertension, stated that the maternity ward’s atmosphere (practicing students, lack of privacy, frequent vaginal examinations, etc.) frightened them and made them to choose CS for delivery as a means to bypass the labour ward [124, 127].

However, some women with previous childbirth were more likely to be in favour of vaginal delivery [109, 112].

### Women’s resources

#### Women’s preferences informed by availability (i.e. what they or insurance can pay)

Supplemental insurance plans in private hospitals support elective CS by providing high-quality facilities for women: “If you want to choose CS, you have to choose private hospitals; you cannot do a CS at governmental hospitals. If you pay more to private hospitals, they provide you with high-quality healthcare” [119].

Women who were not covered by these supplemental insurance plans could not pay for CS, and they “had to” have vaginal delivery [112].

### Health professional factors

#### Health professionals’ beliefs

##### CS is now safe/r option for birth

Some doctors genuinely believed that CS was the better choice for both women and their babies [117, 121, 124, 131]: “VB causes pelvic floors dysfunctions, but CS doesn’t bring this problem” [131] or: “You can travel by a horse, and you can travel by airplane. I think vaginal delivery is like travelling by horse. They tell us that our CS rate is higher than in Europe. So, when my sister had a vaginal delivery in Belgium, they almost killed her. She had severe pain for 24 h. It was such a terrible experience that she came to Iran to have a CS for her second child. We should not listen to these things. The reality is that the CS is faster, better, and I think with new methods, it is even safer for children and women” [124]. Unpredictable status of vaginal delivery and safety of babies were frequently stated reasons by doctors [111, 121, 124].

##### Convenience of birthing to time (work scheduling)

Some doctors stated that the process of vaginal delivery is time-consuming and unpredictable [109, 111, 114, 117] and disturbs night sleeps [111]. They believed that they are too busy to spend time on vaginal delivery [111, 117]: I don’t care about the tariff (the estimated price of services provided.); I don’t have time for it (vaginal delivery). This is of great concern for me as the procedure (vaginal delivery) is time consuming. “ I won’t do it (vaginal delivery), even if I’m paid ten times more [111].

##### Patient pushes the doctor to do CS

Doctors believed that a reduced fertility, as well as the increased age of marriage and pregnancy, was leading to the families’ higher tendencies towards undergoing CS. Some doctors stated that one of the factors affecting the rise in CS is that the women and their families asked for a CS and pushed the doctor to do CS [111, 117, 124]: “Many mothers insist on undergoing a CS from the beginning of their pregnancy” [111]. They believed that it is the right of women to choose what they want [117, 124].

##### Legal issues

Some of the explanations made by the doctors showed the importance of legal matters in increasing the rate of CS [111, 117, 121, 132]: “Being brought to court, even once, make the doctor and her near friends keep away from vaginal deliveries forever. In the court, they behave rudely towards the doctor” [111]. One of the specialists stressed that: “a patient can file a complaint with three officials including medical council, forensic medicine and a special court in the judiciary, making the doctor’s condition worse. The family and the child can also complain to the deliverer even years after the labour, a situation which intensifies the doctors' concerns in this regard” [111].

Doctors stated that there were no guidelines or scientific basis, which would guarantee the judging process [111]. They believed that policies and laws affect the behaviour of healthcare providers [111, 121, 132].

Some doctors claimed that many families receive a certain amount of money from the doctor to withdraw the legal process, stressing that it not only reduces the doctors’ tendency towards performing a vaginal delivery but also tempts other families to file similar complaints [111].

In one study, some midwives were also in favour of CS “whenever a minor problem occurs as they are dealing with feelings of job insecurity. They are afraid to be taken to court for problems caused during vaginal delivery. The law does not protect midwives. Doctors are more protected by law” [111].

#### Financial drivers, financial means and burdens

##### Vaginal delivery fees not worth the time paid for it

A financial incentive in terms of higher fees for doctors in doing CS in private hospitals was considered to be a factor increasing the CS rate [117, 121, 124, 132]: “The CS is faster and easier with more income. I can’t say that all the doctors are completely ignorant of these facts and decide just based on the indications” [124]. Some doctors also claimed that the fee paid for vaginal delivery is not worth the time consumed and stress endured during such a procedure [111].

Changing the tariff imposed on vaginal delivery is one of the strategies adopted by the policymakers to reduce the CS rate. Although in 2004, the Ministry of Health posted a circular defining number of indications for CS, the limitations imposed on paying for CS by insurance companies did not reduce the amount of CS. Industrial relations between patients and doctors, which forced doctors to get paid by patients rather than the insurance company, was one of the main reasons. Moreover, some doctors documented an idea accepted by the insurance company in patients’ files, making an accurate assessment of the underlying reason for CS rather impossible [111]. Specialists have controversial opinions regarding the effect of such a change on the CS rate. Some doctors believed the tariff imposed on vaginal delivery should be two, three or even five times higher than that of CS. Many of them, however, did not think increasing the tariff would solve the problem [111].

#### Communication between women and HCPs

##### Lack of respectful, dignified, and supportive communication with women

Women stated that disrespect, poor communication between them, their families and health professionals and mistreatment could result in deciding not to go for vaginal delivery [27, 111, 112, 114, 115, 119, 120, 123, 125, 127, 128, 131, 132]. Some women had terrible experiences about mistreatment during labour that inhibited them from going back to the labour ward for the next delivery [118, 124]: “They did not behave fairly. They did not allow us to drink water. One of the staff was very bad-tempered. They did not meet our needs” [118]. However, some had a good experience from healthcare providers’ communication and approach [118].

Healthcare providers also confirmed this issue and believed that the work burden did not allow them to have proper communication with women: “The companion talks with the patient and this reduces the patient’s stress. They go to the next step together gradually. But because we don’t have enough human resources in the field, the quality of communication between the midwife and the mother has declined.”[111].

##### Lack of adequate information support

There are so many unknowns surrounding the phenomenon of labour. Women themselves are in the dark regarding what happens during labour. Women’s imprecise knowledge about different delivery methods, their complications, and their hospitalization period has reduced their tendency toward undergoing a vaginal delivery [27, 110, 111, 114, 121]. One midwife said: “evidence-based medicine, which we are trying to follow in our practice, stresses that one of the vaginal delivery complications is relaxation, but do we inform our patients about the complications associated with CS as well? Never. Do we inform mothers about possible side effects of the anesthetic agents, injuries to the genitourinary system, more bleeding, higher infection rates and more infant-related problems associated with CS?”[111].

Both women and healthcare providers believed that providing maternity preparation classes and hotlines could help women to make proper decisions and made them ready for a vaginal delivery and reduce their stress [111, 112, 114, 121, 122].

##### Mistrust

Some women described the level of trust in their doctor as a factor in choosing their method of childbirth [27, 119, 132]. Some stated that they did not trust the recommendations made by their doctors [125], resulting in confusion about making right decision.

Some doctors also stated that the patients did not trust them, and in case of complications, patients saw it as doctors’ fault: “If the sutures were infected, they don’t may be caused due to my obesity. They see it as the doctors’ fault” [124]. A doctor stated that: “lack of trust in the doctors’ accuracy and on-time decision making is another factor forcing mothers to undergo a CS. As a result, we should reassure mothers that C-section would be performed if needed, adding that vaginal delivery would not be our choice if its risks outweigh its benefits. In other words, we choose the method which is best for both the mother and baby.”[111].

#### Healthcare providers’ training, skills, experience, competence, accessibility, number, motivation, and influence

##### Lack of skilled and experienced doctors/midwives during labour and vaginal birth

Many healthcare providers believed that the skills and experience of doctors and residents in conducting a vaginal delivery had been reduced in recent years due to poor quality of education [27, 111, 114]. They believed that because of the reduced number of birth rates in recent years, residents had rare opportunities to do vaginal deliveries.

### Health organization, facility, or system factors

#### Standards of care in birth facilities

##### Physical condition of birth facility (comfortable, calming, clean birth environment)

Poor quality care for women and their children during labour was the most commonly cited external barrier for vaginal delivery [27, 109, 115, 118, 119, 121, 125]. Poor quality environmental facilities, lack of proper equipment, and crowding of birth facilities were also cited [114, 115, 120].

Doctors and midwives also believed that the physical environment of labour rooms was far from standard. This unsuitable condition would negatively affect the women’s perspective and subsequently her decision regarding the type of delivery [111, 114, 121, 124, 129, 131]: “Labour rooms should be equipped with clean restrooms and baths, so that expectant mothers can take a bath whenever they need to. There are no pillows in our department. It is not possible to promote physiologic delivery without spending on it. In our department, restrooms are placed at the other side of the department; the patient is forced to use the basin in front of others, a disgracing condition.”[111]. A midwife stated that the standards of labour rooms have changed over time to reduce the rate of vaginal delivery [111]: “Contrary to international standards, the size of our labour rooms has reduced and they have been converted into operating rooms over time.”[111]. Healthcare providers believed that the labour rooms should be restructured to make spaces between the labor room and delivery room [111, 114].

##### Physical examination and procedures (asking permission, privacy, painful vaginal examination, unnecessary vaginal examinations/interventions)

Some healthcare providers considered the early admission of women as a reason for unnecessary interventions, and consequently CS:“An expectant mother who is being monitored is confined to bed, and this makes her intolerant. She is receiving IV-solutions, and so is not permitted to go to the restroom as she is catheterized. These unnecessary interventions increase the risk of C-section.”[111]. Some of the midwives added that induction of labour in patients with no evidence-based indication might also increase the CS rate, “Induction is equal to increased C-section rate.” [111]. The majority of doctors claimed that medicalizing the process of labour and adding interventions (such as hospitalizing, maintaining an IV-line and injecting solutions, elective induction and frequent vaginal examination) are among the factors turning physiologic labour into a non-physiologic process and consequently increasing the CS rate [111].

Women also stated that they disliked frequent painful vaginal examinations [27, 109, 110, 121, 123, 125, 129, 131] and other approached such as fetal heart rate monitoring during labour [119].

Lack of privacy and shame were other barriers influencing women’s decisions on the mode of delivery [110, 118, 121].

##### Continuous, organized, timely care

Fear of being alone during birth encompassed feelings of loneliness, being ignored by care providers, and feelings of helplessness were common fears expressed by women [27, 121–123].

Doctors also believed that the absence of an on-call doctor as an obstacle in the way of performing vaginal [124]: “the deficiencies of on-call doctors in big cities such as Tehran, where there are long distances between houses and hospitals and there is always fear of traffic and being late, have made doctors perform more C-sections. The presence of an ‘on-call’ doctors in the labour department, therefore, is needed” [111]. Having continuousmidwifery care was proposed by some midwives [114] as a solution to provide more continuous care.

##### Limited availability of pain relief procedures

Both doctors and women believed that providing a comfortable condition might hasten the tendency of vaginal delivery [27, 112, 114, 118, 120, 124, 129]: “we should have epidural anesthesia, which provides the delivery without pain. I don’t know why they don’t use it for all the patients” [124].

##### Lack of partner/family companion during labour/delivery

Midwives or other healthcare providers are the women's only source of support during labour and childbirth because pregnant women are not allowed to have family companion during labour and birth in Iran. Having companions for women during labour and childbirth was mentioned by both women and healthcare providers as a supportive factor [27, 111, 120, 124, 128, 132]: “presence of a companion during the labour and treating mothers nicely can also help tackle the obstacle in this regard” [111].

##### Lack of practical birth guidelines and collaborative midwife-obstetrician models of care

The absence of a scientific and accurate hospital protocol has also contributed to the addition of unnecessary and often non-scientific interventions to the labour process [27, 111]. Another problem mentioned by the majority of the participants was the absence of a precise job description for the specialists and midwives during the labour process. In the absence of such a guideline, it is not clear when the doctor should take responsibility for the operation and who is to blame if and when a problem occurs.

Some specialists stressed that developing a job description for the midwives has various benefits, as it boosts teamwork in the labour process. They, however, added that while sharing the responsibilities is essential for achieving the final goal, it should be done based on scientific evidence and concerns about economic issues. In this regard, some specialists believed that a midwife should perform vaginal birth under the supervision of a specialist. One of the midwives stressed that involving the midwives in the labour process and encouraging teamwork can help reduce the C-section rate [111].

Team working culture and leadership behaviourinfluenced the performance of healthcare providers. Obstetricians and midwives are considered as the two primary arms of the delivery process, but unfortunately, they do not cooperate ideally with each other [111].

#### Communication between doctors and midwives

##### Too little value placed on midwifery care

There have been changes in the professional roles of midwives and doctors during childbirth [27, 111, 119]. Midwives, who previously managed vaginal birth and play a critical role in promoting physiologic labour, have lost their authority; and have faced challenges in realizing their role during birth. Midwives, who used to provide prenatal care at public healthcare centres, can no longer be actively involved in labour and childbirth due to established residency system in most of labour wards of hospitals [119]. Midwives and midwifery students account for less active involvement in vaginal delivery, and subsequently, a decline in the quality of their education has been occurred [111]. One of the midwives believed that the midwives and doctors failed to collaborate due to discrepancies found between their scientific evidences [111].

The role of ambiguity and lack of supervisory are the main problems. One doctor stated: “The midwives are a great help, and they are better in vaginal deliveries, but they should take the responsibility. If they start the delivery, and then call us in a severe condition and put the responsibilities to us, I prefer to have a delivery from the beginning by myself’’ [124].

Residents need to perform a certain number of procedures before graduation. A midwife claimed that: “Many first-year residents transfer women from labour rooms for a C-section as they need to learn C-section before entering the second year” [111]. This was stated as a unique challenge for midwives: “My doctor colleagues try to dominate the whole delivery process, undermining the role of the midwives, who should be responsible for the whole process. If you ask any of the midwives in our hospital, they attest that they have not conducted a natural delivery for years” [111].

##### Financial and legal conflicts

Many midwives claim that doctors receive the money so why should a midwife spend long hours in the labour room; doctors, on the other hand, claim they should earn more money as they are in charge of any possible legal problems linked to labour [111]. On the other hand, insurance companies pay to doctors who are present during labour and delivery. Unless they will not be paid, and the long-time spending with labouring women do not become worse for doctors [121]. One of the specialists added that “Trust issues between the midwives and specialists are a source of defect in the system” [111].

Other midwives stressed that the fact that the midwives are not actively involved in vaginal delivery has contributed to such legal problems. Midwives believe they are not supported by law. One of the doctors noted that during a vaginal birth, midwives decide that performing a C-section is inevitable often too early and without sufficient evidence and as this claim is recorded in the patient’s medical record, the doctor is afraid to give the mother more time to deliver her baby physiologically [111].

## Discussion

This mixed-methods systematic review reported the prevalence of and reasons for women’s, family members’, and health professionals’ preferences for CS in Iran. We included 65 quantitative and 26 qualitative papers in the review, mostly conducted in the urban areas. The quantitative meta-analysis showed that about 5% of nulliparous women preferred CS. The rate was significantly higher among multiparous women (53.05%). Using the qualitative synthesis to help explain why this difference exists between the nulliparous and multiparous women, it may cause by negative experiences of women with first vaginal birth; and preference for CS for the second birth. The majority of women have stated that the reason for the preference of CS was fear of VB. The qualitative meta-synthesis identified that the preference of CS in Iran was influenced by three core themes, including: ‘Women’s factors’, ‘Health professional factors’, and ‘Health organization, facility, or system factors’.

### Unnecessary CS has been rapidly increased in different regions of the globe

Iran has one of the highest CS rates among the Middle Eastern countries. This increase is caused by multiple individuals, facility-level, and system-level factors [27, 111]. Our review showed that pain-related fear of vaginal birth was the most cited individual-level reason for preferring CS. Other reviews of studies conducted in other parts of the world support our review results [22]. Our meta-synthesis also showed that most Iranian women have a deep-rooted fear of labour pain and vaginal birth. Pang et al. also showed that fear of labour pain was the main determinant of birth preference in China [134]. Women’s experience and mistrust of staff were among important facility-level factors, and legal issues were important factors within system-level factors.

Meta-synthesis of the qualitative studies also showed that the barriers and shortages in the health system made women prefer CS. This is supported by several other studies in Iran and other countries [27, 135]. These results showed the importance of multifaceted interventions including educational interventions targeted at women (provision of information, about the risks and benefits of both vaginal delivery and cesarean section), health-care professionals (preserving women’s dignity; interaction between women and providers), and health organizations, facilities or systems (standards of care in facilities, policies and protocols on pain relief for vaginal birth); as proposed by the World Health Organization [38] and reported by several other studies [27, 111, 136].

Our review showed that the proportions of preference for CS were higher in multiparous women. This result was consistent with the results of studies in other countries [22, 137]. Mazzoni et al. 0.2011 showed in their review that the proportion of multiparous women’s preference for CS was 17.5% across Latin and North America, Europe, Asia, and Africa [137]. This result might be attributed to women's negative experience of vaginal delivery [27].

The results of subgroup analysis based on the time of pregnancy showed that fewer women in the third trimester of pregnancy prefer to undergo CS in comparison with the early pregnancy. This may be true because of the impact of antenatal care education. A systematic review conducted by Long et al. has also indicated that women’s preferences changed as the pregnancy progressed, and ambivalence about birth mode was evident [22]. Meanwhile, women also make their decision based on other factors, such as their experiences, information that they receive from the most important people, and environmental factors [135].

Our qualitative evidence indicated that the financial drivers could encourage doctors to do CS without clinical indication for CS. This finding resonates with broader literature reporting women’s and health professional’s views of the reasons behind CS rates [111]. Although, despite the structural reforms, including free of charge vaginal delivery in public hospitals, the rate of CS still is high [24].

### Strengths and limitations of the review

This review is the first mixed-method review of its kind in Iran that brings together the evidence of stakeholders’ perspectives on preferences on mode of delivery. In this review, we captured all stakeholders’views, including women, family members, health professionals, and health administrators, on the preference and reasons for CS. We included both English and Persian studies based on the abilities of the review team. There were some limitations to this review. The heterogeneity was high, similar to that reported in other previous meta-analysis of women’s preference for CS [22, 137]. In the included primary studies, most of the risk level was unknown, and most of the participants were pregnant women, regardless of parity. The qualities of some Persian publications were appraised as low, using reporting standards standards, which may threaten the confidence in the evidence.

### Implications for practice and future research

The findings of this review suggest that there are several reasons behind the high rate of CS in Iran that is not necessarily along with the women’s requests for CS based on preference for this mode of delivery. These reasons should be clearly defined, and multifaceted strategies targeting women, health professionals, and healthcare systems should be designed and implemented.

Although there has been an overall improvement in maternal and reproductive health in Iran since 1990, there are still challenges facing the country about maternal health improvement, including implementing standard clinical protocols for providing pregnancy, delivery and post-delivery services and promoting the quality of reproductive health services (138). Developing national guidelines and culture-oriented frameworks to decrease unnecessary CS is suggested.

The proportion of CS is higher in private hospitals. Financial incentives for VBs in private hospitals could help to decrease the CS rate. Developing availability of and strategies for a vaginal birth after cesarean section and training the professionals can provide a great potential to reduce the number of CSs.

Research studies should be conducted to identify local barriers and right strategies embedded in health systems towards optimizing the use of CS, and planning and implementing intervention strategies which can be assessed through randomized controlled trials. We have appraised the quality of the included studies to provide the level of confidence for the review findings. This assessment showed us that most of the included articles had some methodological limitations (for example: quality of analysis). Conducting robust and precise studies can help to have more reliable resultsand an actionable evidence base.

## Conclusion

Our review showed a series of multiple individuals, health facilities, and health system factors on the preference for CS. Numerous attempts were made in recent years to design, test and implement interventions to decrease unnecessary CS in Iran, such as the mother-friendly hospitals; the development of standard protocols for labor and birth; implementation of preparation classes for women, midwives, and gynecologists; and workshops for specialists and midwives through the “health sector evolution policy”. Although these programs were effective, the rate is still high, and other non-clinical initiatives might be helpful and needed to reduce unnecessary CS rates.

## Supplementary information

**Additional file 1: Table S1.** Search Strategies

**Additional file 2: **Data extraction form.

**Additional file 3: **Completed data extraction (quantitative papers).

**Additional file 4: **Text S1. Quality Assessment Prompts for Quantitative Studies.

**Additional file 5: **Characteristics of the included studies.

**Additional file 6: **Meta Regression Result.

**Additional file 7: **Meta Regression Result.

**Additional file 8: **Reasons for preference for caesarean section reported by women, quantitative studies.

**Additional file 9: **Qualitative assessment.

**Additional file 10: **Tables 4–29 theCERQual findings.

**Additional file 11: **Summaries of initial concepts, emergent themes, final themes, and supporting quotes.

## Data Availability

Not applicable. This manuscript does not contain any data, as this is a study design manuscript.

## Abbreviations

CS: Cesarean section
VB: Vaginal birth
WHO: World Health Organization
