# Supplementary material for: Prevalence of and reasons for women’s, family members’, and health professionals’ preferences for cesarean section in Iran: a mixed-methods systematic review
Source: Reprod Health. 2021 Jan 2;18:3. doi: 10.1186/s12978-020-01047-x (PMC7778821; doi:10.1186/s12978-020-01047-x)
Supplement: Supplementary file 1 — Additional file 1: Table S1. Search Strategies [file 12978_2020_1047_MOESM1_ESM.docx]

**Pubmed / Medline**

**http://www.pubmed.gov**

| # | Searches | Results | October2018 -10/08/2019 | 1990-2019 |
| --- | --- | --- | --- | --- |
| 1 | "Cesarean Section"[Mesh] OR Cesarean [TIAB] OR Caesarean [TIAB] OR “C-sections” [TIAB] OR “C-section”[TIAB] OR “Abdominal Deliveries”[TIAB] OR “Abdominal Delivery”[TIAB] OR Postcesarean [TIAB] OR Postcaesarean [TIAB] OR “Post cesarean” [TIAB] OR “Post caesarean” [TIAB] | 59563 | 3205 | 55489 |
| 2 | "Patient Preference"[Mesh] OR "Nurse-Patient Relations"[Mesh] OR "Interviews as Topic"[Mesh] OR "Emotions"[Mesh] OR "Elective Surgical Procedures"[Mesh] OR "Unnecessary Procedures"[Mesh] OR "Physician-Patient Relations"[Mesh] OR "Patient Satisfaction"[Mesh] OR "Natural Childbirth"[Mesh] OR "Health Knowledge, Attitudes, Practice"[Mesh] OR "Choice Behavior"[Mesh] OR "Attitude to Health"[Mesh] OR "Cesarean Section/psychology"[Mesh] OR "Culture"[Mesh] OR "ethnology" [Subheading] OR "Vaginal Birth after Cesarean"[Mesh] OR “decision making” [MH] OR “pain/psychology” [MH] OR “Delivery, obstetric/psychology” [MH] OR “pregnant women/psychology” [MH] OR Patient Participation[Mesh] OR Fear* [TIAB] OR preference* [TIAB] OR prefers [TIAB] OR preferred [TIAB] OR decision* [TIAB] OR “non medical factors” [TIAB] OR “non medical indications” [TIAB] OR option [TIAB] OR options [TIAB] OR optional [TIAB] | 1520832 | 90073 | 1740704 |
| 3 | Step 1 and Step 2 | 7793 | 461 | 9516 |
| 4 | Publication date from 1990/01/01 to 2050/12/31 | 7191 |  |  |
| 5 | #3 and Iran |  | 13 | 136 |

**PsycINFO**

**http://search.ebsco.com**

| # | Searches | Results | October2018 -10/08/2019 | 1990-2019 |
| --- | --- | --- | --- | --- |
| 1 | DE "Caesarean Birth" OR TI Cesarean OR TI Caesarean OR TI (C-sections) OR TI(C-section) OR TI (Abdominal Deliveries) OR TI (Abdominal Delivery) OR TI Postcesarean OR TI Postcaesarean OR TI (Post cesarean) OR TI (Post caesarean) OR AB Cesarean OR AB Caesarean OR AB (C-sections) OR AB (C-section) OR AB (Abdominal Deliveries) OR AB (Abdominal Delivery) OR AB Postcesarean OR AB Postcaesarean OR AB (Post cesarean) OR AB (Post caesarean) | NA | 11 | 166 |
| 2 | DE "Preferences" OR DE "Decision Making" OR DE "Emotional States" OR TI Fear* OR TI preference* OR TI prefers OR TI preferred OR TI decision* OR TI (non medical factors)   OR TI (non  medical indications) OR TI option  OR TI options  OR TI optional OR AB Fear* OR AB preference* OR AB prefers OR AB preferred OR AB decision* OR AB (non medical factors)   OR AB (non  medical indications) OR AB option  OR AB options  OR TI optional | NA | 1,083 | 15,668 |
| 3 | Published Date: 19900101-20161231 | 269 |  |  |
| 4 | #1 and #2 |  | 1 | 6 |
| 5 | #4 and Iran |  | 0 | 0 |

**CINAHL**

**http://search.ebsco.com**

| # | Searches | Results | October2018 -10/08/2019 | **2019-1990** |
| --- | --- | --- | --- | --- |
| 1 | (MH "Cesarean Section+") OR TI Cesarean OR TI Caesarean OR TI (C-sections) OR TI(C-section) OR TI (Abdominal Deliveries) OR TI (Abdominal Delivery) OR TI Postcesarean OR TI Postcaesarean OR TI (Post cesarean) OR TI (Post caesarean) OR AB Cesarean OR AB Caesarean OR AB (C-sections) OR AB (C-section) OR AB (Abdominal Deliveries) OR AB (Abdominal Delivery) OR AB Postcesarean OR AB Postcaesarean OR AB (Post cesarean) OR AB (Post caesarean) | NA | 11 | 166 |
| 2 | (MH "Professional-Patient Relations+") OR (MH "Interviews+") OR (MH "Surgery, Elective+") OR (MH "Unnecessary Procedures") OR (MH "Attitude to Health+") OR (MH "Alternative Birth Methods+") OR (MH "Home Childbirth") OR (MH "Prepared Childbirth") OR (MH "Culture+") OR (MH "Ethnological Research") OR (MH "Vaginal Birth+") OR (MH "Health Knowledge") OR (MH "Decision Making, Patient") OR (MH "Decision Support Techniques") OR (MH "Decision Making, Family") OR (MH "Pain/PF") OR (MH "Expectant Mothers/PF") OR (MH "Delivery, Obstetric/PF") OR TI Fear* OR TI preference* OR TI prefers OR TI preferred OR TI decision* OR TI (non medical factors)   OR TI (non  medical indications) OR TI option  OR TI options  OR TI optional OR AB Fear* OR AB preference* OR AB prefers OR AB preferred OR AB decision* OR AB (non medical factors)   OR AB (non  medical indications) OR AB option  OR AB options  OR TI optional | NA | 1,079 | 15,506 |
| 3 | Published Date: 19900101-20161231 |  |  |  |
| 4 | Exclude MEDLINE records | 356 |  |  |
| 5 | #1 and #2 |  | 1 | 6 |
| 6 | #5 and Iran |  | 0 | 0 |

**EMBASE**

**http://www.embase.com**

| # | Searches | Results | October2018 -10/08/2019 | **2019-1990** |
| --- | --- | --- | --- | --- |
| 1 | 'cesarean section'/exp OR 'cesarean section' OR 'cesarean section kit'/exp OR 'cesarean section kit' OR cesarean:de,ab,ti OR caesarean:de,ab,ti OR 'c-sections':de,ab,ti OR 'c-section':de,ab,ti OR 'abdominal deliveries':de,ab,ti OR 'abdominal delivery':de,ab,ti OR postcesarean:de,ab,ti OR postcaesarean:de,ab,ti OR 'post cesarean':de,ab,ti OR 'post caesarean':de,ab,ti | 90871 | 11,756 | 98,706 |
| 2 | 'patient preference'/exp OR 'nurse patient relationship'/exp OR 'interview'/exp OR 'emotion'/exp OR 'elective surgery'/exp OR 'unnecessary procedure'/exp OR 'doctor patient relation'/exp OR 'patient satisfaction'/exp OR 'natural childbirth'/exp OR 'attitude to health'/exp OR 'cultural anthropology'/exp OR 'ethnology'/exp OR 'patient participation'/exp OR fear*:de,ab,ti OR preference*:de,ab,ti OR prefers:de,ab,ti OR preferred:de,ab,ti OR decision*:de,ab,ti OR 'non medical factors':de,ab,ti OR 'non medical indications':de,ab,ti OR option:de,ab,ti OR options:de,ab,ti OR optional:de,ab,ti OR (('vaginal birth' OR 'vaginal births') NEAR/2 (cesarean OR caesarean)):de,ab,ti OR ('pregnant woman' NEAR/10 psycholog*):de | 1936849 | 274,415 | 2,401,648 |
| 3 | Step 1 and Step 2 | 12479 | 2,216 | 17,062 |
| 4 | [1990-2016]/py | 11753 |  |  |
| 5 | #3 and Iran |  | 58 | 382 |

**Popline**

[**http://www.popline.org**](http://www.popline.org)

| # | Searches | Results | October2018 -10/08/2019 | 1990-2019 |
| --- | --- | --- | --- | --- |
| 1 | ((Cesarean ) OR (Caesarean ) OR (Cesareans ) OR (Caesareans ) OR (C-sections) OR (C-section) OR (Abdominal Deliveries) OR (Abdominal Delivery) OR (Postcesarean) OR (Postcaesarean)) AND ((Fear) OR (FEARS) OR ( preference) OR (preferences) OR (prefers) OR (prefer) OR (preferred) OR (decision) OR (decisions) OR (non medical factors)   OR (non  medical indications) OR (option)  OR ( options) OR (optional)) | NA | 16 |  |
| 2 | Published Date: 1990-2016 | 414 |  |  |
| 3 | #1 and Iran |  | 2 |  |

**Global Health Library - Global Index Medicus**

[**http://www.globalhealthlibrary.net/php/index.php**](http://www.globalhealthlibrary.net/php/index.php)

**(Indexes - Regional Indexs) Option** **selected.**

| # | Searches | Results | October2018 -10/08/2019 | 1990-2019 |
| --- | --- | --- | --- | --- |
| 1 | ((Cesarean ) OR (Caesarean ) OR (Cesareans ) OR (Caesareans ) OR (C-sections) OR (C-section) OR (Abdominal Deliveries) OR (Abdominal Delivery) OR (Postcesarean) OR (Postcaesarean)) AND ((Fear) OR (FEARS) OR ( preference) OR (preferences) OR (prefers) OR (prefer) OR (preferred) OR (decision) OR (decisions) OR (non medical factors)   OR (non  medical indications) OR (option)  OR ( options) OR (optional)) | NA | 323 | 682 |
| 2 | Published Date: 1990-2016 | 251 |  |  |
| 3 | #1 and Iran |  | 2 | 20 |

**Search update in 2018**

*English databases 1/1/2017-18/4/2018 (With duplicates removed)**

| Database | Results |
| --- | --- |
| PubMed / Medline | 282 |
| PsycINFO | 17 |
| CINAHL | 165 |
| EMBASE | 2908 |
| Popline | 16 |
| Global Health Library - Global Index Medicus (GIM) | 52 |
| Total | 3440 |

*With duplicates, total = 4,386
