# Supplementary material for: Prevalence of and reasons for women’s, family members’, and health professionals’ preferences for cesarean section in Iran: a mixed-methods systematic review
Source: Reprod Health. 2021 Jan 2;18:3. doi: 10.1186/s12978-020-01047-x (PMC7778821; doi:10.1186/s12978-020-01047-x)
Supplement: Supplementary file 4 — Additional file 4: Text S1. Quality Assessment Prompts for Quantitative Studies. [file 12978_2020_1047_MOESM4_ESM.docx]

**S1 Text Quality Assessment Prompts for Quantitative Studies**

1. **General**
2. Objetives/Aims
3. Objectives/aims are clearly stated
4. Objectives/aims are partially/poorly stated
5. Objectives/aims are not stated
6. **Methodology and data collection**
7. Population
   1. The eligibility criteria is clear/population clearly defined
   2. The eligibility criteria is not clear/population not clearly defined
8. Representativeness/Recruitment
9. Truly representative of the average in the target population (all subjects or random sampling)
10. Somewhat representative of the average in the target population (non-random sampling)
11. Selected group of users
12. No description of the sampling strategy or unclear
13. Sample size
14. All subjects in the target population (n≥100) OR explanation regarding sample size used (regardless of n)
15. No explanation regarding sample size used, unsatisfactory size (n<100) or unclear
16. Non-respondents
17. Comparability between respondent and non-respondent characteristics are established, and the response rate is satisfactory (≥70%)
18. The response rate is unsatisfactory, or the comparability between respondents and non-respondents is unsatisfactory (<70%)
19. No description of the response rate or the characteristics of the responders and the non-responders or unclear
20. Data collection tool
21. Description of tool (e.g. questionnaire) and development or source thereof used to assess the outcome(s) is/are clearly defined
22. Description of tool (without development or source) used to assess the outcome(s) is/are clearly defined
23. Description of tool and/or development thereof used to assess the outcome(s) is poorly, unclearly or not defined
24. Outcome-related Questions and /or Statements
25. The questions and/or statements pertaining to the outcome were stated explicitly, clearly, are unambiguous and are not phrased with bias towards any particular view
26. The questions and/or statements pertaining to the outcome were not stated explicitly, clearly or they are ambiguous or were phrased with bias towards any particular view
27. Ethical considerations: Compliance with professional and ethical standards
28. Informed consent was obtained from participants and ethical approval of study was obtained (if applicable)
29. Unclear/not reported if informed consent was obtained from participants OR unclear/not reported if ethical approval was obtained (if applicable)
30. Unclear/not reported if informed consent was obtained from participants AND unclear/not reported if ethical approval of study was obtained (if applicable)
31. **Data presentation and analysis**
32. Clarity
33. Data is presented with utmost clarity including numerators, denominators and missing
34. At least some data is unclear requiring guesswork or leaving questions unanswered.
35. Consistency
36. There is consistency between the research question, data available and reporting of data
37. At least some inconsistencies between the research question, data available and reporting of that data
