# Supplementary material for: Prevalence of and reasons for women’s, family members’, and health professionals’ preferences for cesarean section in Iran: a mixed-methods systematic review
Source: Reprod Health. 2021 Jan 2;18:3. doi: 10.1186/s12978-020-01047-x (PMC7778821; doi:10.1186/s12978-020-01047-x)
Supplement: Supplementary file 5 — Additional file 5: Characteristics of the included studies. [file 12978_2020_1047_MOESM5_ESM.docx]

S3 Table Characteristics of the included studies

| n | Study name | | Language | Participants | Time point | Study design | Region | Location | Facility/population-based | Number of settings | Data collection method | Year of data collection | Quality assessment | Quality assessment rank |
| --- | --- | --- | --- | --- | --- | --- | --- | --- | --- | --- | --- | --- | --- | --- |
| Quantitative studies | | | | | | | | | | | | | | |
| 1 | | Rajabi et al 2016 | English | Pregnant women (n=2199, parity unknown) | T2 | longitudinal | Fars | Not reported | Facility | Not reported | Self-administered | 2012-2013 | 8 | high |
| 2 | | Dehghani et al 2014 | English | Pregnant women (n=300, parity unknown) | Any | Cross-sectional | Teheran | Urban | Facility | 2 | Self-administered | 2009 | 9 | high |
| 3 | | Gholami et al 2014 | English | Pregnant women with previous CS(n= 292) | Unknown | Cross-sectional | Neyshabur | Not reported | Unknown | Not reported | Self-administered | 2011 | 7.5 | high |
| 4 | | Maharlouei et al 2013 | English | Pregnant women (n=6921, parity unknown) | T1 and T2 | Cross-sectional | Fars | Mixed | Population | Not reported | Self-administered | 2011-2012 | 6.5 | low |
| 5 | | Eynsheykh et al 2013 | English | Pregnant women (n=, parity unknown) | Unknown | Cross-sectional | Savojbolagh province | Not reported | Facility | 4 | Self-administered | Not reported | 2 | low |
| 6 | | ChoobMasjedi et al 2012 | English | Pregnant women without previous CS(n= 300) | T2 | Cross-sectional | Tehran | Urban | Facility | 2 | Self-administered | 2010 | 7 | middle |
| 7 | | Shakeri et al 2012 | English | Nulliparous pregnant women(n= 697) | T1 and T2 | Cross-sectional | Shakeri | Urban | Facility | 2 | Not reported | Not reported | 2 | low |
| 8 | | Matinnia et al 2015 | English | Nulliparous pregnant women(n= 342) | T1 | Cross-sectional | Unknown | Urban | Facility | 4 | Not reported | Not reported | 7.5 | high |
| 9 | | Navaee et al 2015 | English | Nulliparous pregnant women(n= 67) | T2 | cross-sectional | Three cities in Mashad province | Urban | Facility | 10 | Not reported | Not reported | 6.5 | low |
| 10 | | Gholami et al 2013 | English | Nulliparous pregnant women (n= 797) | Any | cross-sectional | Neyshabur | Not reported | Facility | Not reported | Self-administered | 2011 | 7.5 | high |
| 11 | | Aali et al 2005 | English | Pregnant women (n=204, parity unknown) | Any | cross-sectional | Kerman | Urban | Facility | 1 | Self-administered | 1999 | 4 | low |
| 12 | | Bakhtari et al 2017 | English | Nulliparous pregnant women(n= 434) | T2 | Cross-sectional | Urmia | Urban | Facility | 36 | Self-administered | 2015 | 5 | low |
| 13 | | Moasheri et al 2016 | Persian | Nulliparous pregnant women(n= 196) | T2 | Cross-sectional | Birjand | Urban | Facility | 4 | Self-administered | 2014 | 9 | high |
| 14 | | Rezakhani Moghaddam et al 2013 | persian | Nulliparous pregnant women(n= 140) | T2 | Interventional | Ardabil | Urban | Facility | 10 | Face-to-face interview | 2011 | 8.5 | high |
| 15 | | Khan-Jeihooni et al 2014 | persian | Nulliparous pregnant women(n=100) | T2 | Interventional | Fasa | Urban | Facility | 4 | Not reported | 2011 | 9 | high |
| 16 | | Atghaee et al 2010 | persian | Pregnant women (n=400, parity unknown) | Unknown | Cross-sectional | Kerman | Urban | Facility | Not reported | Self-administered | 2010 | 8.5 | high |
| 17 | | Abdolkarimi et al 2016 | persian | Pregnant women (n=200, parity unknown) | T1 and T2 | Cross-sectional | Urmia | Rural | Facility | 10 | Face-to-face interview | 2014 | 8 | high |
| 18 | | Payman et al 2010 | persian | Pregnant women (n=390, parity unknown) | T2 | Cross-sectional | Mashhad | Urban | Facility | Not reported | Face-to-face interview | Not reported | 7.5 | high |
| 19 | | Andaroon et al 2017 | persian | Nulliparous pregnant women (n= 220  ) | T2 | Cross-sectional | Mashhad | Urban | Facility | 4 | Face-to-face interview | Not reported | 9 | high |
| 20 | | Moradan et al 2004 | persian | Pregnant women (n=400, parity unknown) | Unknown | Cross-sectional | Semnan | Urban | Facility | Not reported | Face-to-face interview | 2004 | 5 | low |
| 21 | | mohamadpoorasl et al 2009 | persian | Pregnant women (n=250, parity unknown) | Unknown | Cross-sectional | Maraghe | Urban | Facility | 3 | Self-administered | 2006 | 5.5 | low |
| 22 | | Norizadeh et al 2009 | persian | Pregnant women (n=450, parity unknown) | Unknown | Cross-sectional | Marand | Urban | Facility | 9 | Self-administered | 2004 | 6.5 | low |
| 23 | | Amidi et al 2005 | persian | Nulliparous pregnant women(n= 54) | T1 | Interventional | Esfahan | Urban | Facility | 1 | Self-administered | Not reported | 4.5 | low |
| 24 | | Shahraki Sanavi et al 2014 | persian | Pregnant women (n=200, parity unknown) | T2 | Interventional | Zahedan | Urban | Facility | 5 | Face-to-face interview | 2010-2011 | 7.5 | high |
| 25 | | Mohamadi Tabar et al 2009 | persian | Nulliparous pregnant women(n= 618) | Unknown | Cross-sectional | Tehran | Urban | Facility | Not reported | Face-to-face interview | 2007 | 7.5 | high |
| 26 | | Mohammadi et al 2014 | persian | Pregnant women (n=154, parity unknown) | T2 | Cross-sectional | Tehran | Urban | Facility | 9 | Not reported | Not reported | 8.5 | high |
| 27 | | Salehi et al 2014 | persian | Pregnant women (n=305, parity unknown) | Unknown | Cross-sectional | Bane | Urban | Facility | 4 | Not reported | 2014 | 6.5 | low |
| 28 | | Kiani et al 2014 | persian | Midwives(n= 325) | NA | Cross-sectional | Tehran | Urban | Facility | 2 | self and face to face | Not reported | 7.5 | high |
| 29 | | Ziyagham et al 2014 | persian | Nulliparous pregnant women(n= 150) | Unknown | cross-sectional | Ahvaz | Urban | Facility | 8 | self and face to face | Not reported | 7 | middle |
| 30 | | Lashgari et al 2005 | persian | Nulliparous pregnant women(n= 200) | T1 | Interventional | Tehran | Urban | Facility | 1 | Self-administered | Not reported | 7 | middle |
| 31 | | Yousefzade et al 2014 | persian | Nulliparous pregnant women(n= 64) | T1 | Interventional | Mashhad | Urban | Facility | 2 | Self-administered | Not reported | 8 | high |
| 32 | | Bani et al 2010 | persian | Gynecologist (n= 90),  Midwives(n= 153) | NA | cross-sectional | Tabriz | Urban | facility | Not reported | Self-administered | 2009 | 8.5 | high |
| 33 | | Marashi et al 2018 | persian | Nulliparous pregnant women(n= 161) | T1 | cross-sectional | Babol | Urban | Facility | 3 | Self-administered | 2016 | 9 | high |
| 34 | | Rahmati et al 2014 | persian | Pregnant women (n=392, parity unknown) | Unknown | cross-sectional | Tehran | Urban | Facility | 2 | Self-administered | 2012-2013 | 8 | high |
| 35 | | Negahban et al 2006 | persian | Pregnant women (n=256, parity unknown) | T1 and T2 | cross-sectional | Rafsanjan | Urban | Facility | Not reported | Self-administered | Not reported | 6 | low |
| 36 | | Shahbazazdegan et al 2010 | persian | Pregnant women (n=245, parity unknown) | Unknown | cross-sectional | Ardabil | Urban | Facility | 5 | Self-administered | 2009 | 5.5 | low |
| 37 | | Abedian et al 2012 | persian | Nulliparous pregnant women(n= 67) | T2 | Interventional | Mashhad | Urban | Facility | 5 | Not reported | 2010 | 7.5 | high |
| 38 | | Faraji et al 2003 | persian | Pregnant women (n=602, parity unknown) | Unknown | cross-sectional | Rasht | Urban | Facility | 12 | self and Face-to-face interview | 2001 | 6.5 | low |
| 39 | | Faramarzi et al 2001 | persian | Pregnant women (n=250, parity unknown) | Unknown | cross-sectional | Babol | Urban | Facility | 4 | Face-to-face interview | 1999 | 5 | low |
| 40 | | Yassaee et al 2007 | persian | Nulliparous pregnant women(n= 160) | Unknown | cross-sectional | Tehran | Urban | Facility | 2 | Not reported | 2000 | 6 | low |
| 41 | | Moeini et al 2011 | persian | Pregnant women (n=346, parity unknown) | Unknown | cross-sectional | Hamedan | Urban | Facility | 5 | Self-administered and Face-to-face interview | 2006 | 7 | middle |
| 42 | | Afshari et al 2013 | persian | Nulliparous pregnant women(n=104) | T2 | cross-sectional | Semiroum | Mixed | Facility | 7 | Not reported | 2011 | 8 | high |
| 43 | | Besharati et al 2011 | persian | Pregnant women (n=72, parity unknown) | T2 | Interventional | Rasht | Urban | Facility | 6 | Self-administered | 2010 | 9 | high |
| 44 | | Sharifirad et al 2008 | Persian | Nulliparous pregnant women(n=140) | Unknown | Cross-sectional | Khomeiny shahr | Urban | Facility | 8 | Self-administered | Not reported | 6.5 | low |
| 45 | | Aram et al 2002 | Persian | Pregnant women (n=500, parity unknown) | T1 | Cross-sectional | Esfahan | Urban | Facility | Not reported | Self-administered | 2002 | 5.5 | low |
| 46 | | Movahed et al 2012 | Persian | Pregnant women (n=600, parity unknown) | Unknown | Cross-sectional | Shiraz | Urban | Facility | Not reported | Self-administered | Not reported | 7 | middle |
| 47 | | Vafaee et al 2014 | Persian | Pregnant women (n=417, parity unknown)  Partners(n=417) | Unknown | Cross-sectional | Shiraz | Urban | Facility | 3 | Self-administered and Face-to-face interview | Not reported | 7.5 | high |
| 48 | | Heydari et al 2019 | English | Nulliparous pregnant women(n=3940) | Unknown | Cross-sectional | Shiraz | Urban | Facility | 5 | Self-administered and interview | 2016 | 7.5 | high |
| 49 | | Jalali et al 2019 | English | Nulliparous, Multiparous pregnant women(n=3940) | T2 | Cross-sectional | Tehran | Urban | Facility | 1 | Self-administered | 2016 | 8 | High |
| 50 | | Kananikandeh et al 2018 | English | Pregnant women (n=76, parity unknown) | T2 | Interventional | Pars Abad (Ardabil) | Urban | Facility | 4 | Self-administered | 2014 | 10 | high |
| 51 | | Matinnia et al 2018 | English | Nulliparous pregnant women(n=342) | Unknown | Cross-sectional | Hamedan | Urban | Facility | 3 | Self-administered | Not reported | 9 | high |
| 52 | | Rasoli et al 2019 | English | Nulliparous pregnant women(n=211) | T2 | Interventional | Behshahr, Mazandaran | Urban | Facility | 1 | Self-administered | 2015 | 9 | high |
| 53 | | Safari-Moradabadi et al 2018 | English | Nulliparous pregnant women(n=210) | T2 | Cross-sectional | Bandar Abbas | Urban | Facility | 12 | Self-administered | 2015 | 8.5 | high |
| 54 | | Khaledian et al 2018 | Persian | pregnant women(n=79) | Unknown | Cross-sectional | Semnan | Urban | Facility | 1 | Self-administered | 2016-2017 | 7.5 | High |
| 55 | | Makhouli et al 2018 | Persian | pregnant women(n=36) | T1 | Interventional | Esfahan | Urban | facility | 1 | Self-administered | 2016 | 8 | high |
| 56 | | Khavandiaghdam et al2019 | Persian | Nulliparous pregnant women(n=100) | T1 and T2 | Interventional | Ardabil | Urban | Facility | 1 | Self-administered | 2017 | 8.5 | high |
| 57 | | Baghianimoghadam et al2013 | Persian | Nulliparous pregnant women(n=130) | T2 | Cross-sectional | Yazd | Urban | Facility | 6 | Self-administered | 2013 | 9 | High |
| 58 | | Dadipoor et al 2017 | Persian | Nulliparous pregnant women(n=210) | Unknown | Cross-sectional | Bandar Abbas | Urban | Facility | 12 | Self-administered | 2016 | 9 | High |
| 59 | | Najafi- Sharjabad et al 2017 | Persian | pregnant women(n=462) | Unknown | Cross-sectional | Bushehr | Urban | Facility | 7 | Self-administered | 2015 | 9 | High |
| 60 | | Najafi et al 2015 | Persian | Nulliparous pregnant women(n=202) | Unknown | prospective cohort study | Gilan | Urban | Facility | 19 | Self-administered | 2013-2014 | 8.5 | High |
| 61 | | Darsareh et al 2016 | English | pregnant women(n=470) | Unknown | Cross-sectional | Bandar Abbas | Urban | Facility | 7 | Self-administered | 2016 | 9.5 | High |
| 62 | | Masoumi et al 2016 | English | pregnant women(n=150) | Unknown | Interventional | Hamedan | Urban | Facility | Unknown | Self-administered | 2016 | 10 | High |
| 63 | | Zamani-Alavijeh et al 2018 | English | pregnant women(n=200) | Unknown | Cross-sectional | Isfahan | Urban | Facility | 1 | Self-administered | 2018 | 9 | High |
| 64 | | Sharifzadeh et al 2018 | Persian | Nulliparous pregnant women(n=55) | T1 | Interventional | Sabzevar | Urban | Facility | 6 | Self-administered | 2016 | 7.5 | High |
| 65 | | Siabani et al 2019 | English | pregnant women(n=410) | Unknown | Interventional | Kermanshah | Urban | Facility | 9 | Self-administered | 2016 | 9 | High |
| **Qualitative studies** | | | | | | | | | | | | | | |
| 66 | Ahmad Shirvani et al 2014 | | persian | Pregnant women with previous CS or NVD(n=16) | Not reported | Qualitative | Sari | Urban | Health facility based | not reported | In-depth interviews | 2014 |  | C |
| 67 | Javaheri et al 2016 | | persian | postpartum women(delivered by CS, n=15) | Not reported | Qualitative | Tehran | Urban | Health facility based | 1 | Interviews | 2016 |  | D |
| 68 | Rahnama et al 2015 | | persian | Pregnant women(n=36) | Not reported | Qualitative | Tehran | Urban | Health facility based | 5 | Interviews | 2014 |  | C |
| 69 | Shams et al 2016 | | persian | physicians (n=2), midwife (n=10)and Nulliparous Pregnant women (n=37) | Not reported | Qualitative | Boyear ahmad | Mixed | Health facility based | 6 | Semi-structured interviews and Focus group discussions | 2016 |  | C |
| 70 | Shahoei et al 2014 | | persian | Nulliparous Pregnant women Non-medically indicated caesarean section(n=12) | T3 | Qualitative | sanandaj | Urban | Health facility based | not reported | Semi-structured nterviews and In-depth interviews | 2014 |  | C |
| 71 | Vaziri et al 2013 | | persian | Nulliparous women with NVD or CS(n=17) | Not reported | Qualitative | Shiraz | Urban | Health facility based | not reported | Semi-structured nterviews | 2013 |  | B |
| 72 | Hajiyan et al 2010 | | persian | Pregnant women (n=16), physicians, midwife and Anesthesiologist(n=8) | Not reported | Qualitative | Shahroud | Urban | Health facility based | not reported | Semi-structured nterviewsused Delphi method and Focus group discussions | 2010 |  | C |
| 73 | Borghei et al 2016 | | persian | Pregnant women(n=3), husbands (n=3) and midwife (n=3) | Antenatal care | Qualitative | Gorgan | Urban | Health facility based | not reported | semi- stuctured interviews | 2016 |  | B |
| 74 | Darvishi et al 2012 | | persian | Physicians( n= 10), Postpartum women with elective CS(n=9), and Postpartum women with emergency CS(n= 11) | Postpartum women | Qualitative | Ahvaz & Mahshahr | Mixed | Health facility based | not reported | Semi-structured nterviews | 2012 |  | C |
| 75 | Abbaspoor et al 2014 | | persian | Pregnant and postpartum women with NVD or CS (n=18) | Not reported- Postpartum women | Qualitative | Ahvaz | Urban | Health facility based | not reported | semi- stuctured interviews and indepth interviews | 2014 |  | C |
| 76 | Bayrami et al 2011 | | persian | Nulliparous postpartum women with NVD (n= 20) | Postpartum women | Qualitative | Khoy | Urban | Health facility based | not reported | semi- stuctured interviews and indepth interviews | 2011 |  | C |
| 77 | Vedadhir et al 2011 | | persian | postpartum women with NVD or CS experiences(n=16) | Postpartum women | Qualitative | tabriz | Urban | Health facility based | not reported | Interviews | 2012 |  | C |
| 78 | Rahnama et al 2016 | | English | pregnant women(n=36) | Not reported | Qualitative | tehran | Not reported | Health facility based | not reported | in-depth interviews and focus group | 2016 |  | C |
| 79 | Mobarakabadi et al 2015 | | English | pregnant and postpartum women(n= 27) | Not reported- postpartum women (1-10 days) | Qualitative | Mashhad | Urban | Health facility based | not reported | n depth unstructured interviews | 2015 |  | B |
| 80 | Shahoei et al 2014 | | English | pregnant women(n=22) | T3 | Qualitative | Sanandaj | Urban | Health facility based | 3 | semi structured nterviews | 2014 |  | B |
| 81 | Faisal et al 2014 | | English | Nulliparous pregnant women | Not reported | Qualitative | Hamadan | Urban | Health facility based | 4 | in-depth semi - structured interviews | 2013 |  | B |
| 82 | Hajian et al 2013 | | English | pregnant women(n=17) | Not reported | Qualitative | Shahroud | Urban | Health facility based | 6 | focus group discussions | 2013 |  | B |
| 83 | Bagheri et al 2013 | | English | obstetricians and resident medical staffs(n=18) | NA | Qualitative | Kashan | Urban | Health facility based | 3 | semi-structured interviews | 2012 |  | B |
| 84 | Sanavi et al 2012 | | English | pregnant women with the intention or decision to elective CS( n=200) | T3 | Qualitative | Zahedan | Urban | Health facility based | 2 | focus group discussions | 2012 |  | D |
| 85 | Yazdizadeh et al 2011 | | English | healthcare providers( n=4) | NA | Qualitative | not reported | Not reported | Health facility based | not reported | in-depth interviews and document analyses | 2011 |  | A |
| 86 | Latifnejad Roudsari et al 2015 | | English | Midwives(n=7), obstetricians(n=7), and non-pregnant women(n=9), pregnant women(n=12), postpartum women(n=10) | NA | Qualitative | Tonekabon | Urban | Health facility based | not reported | Semi-structured nterviews and articipant observations | 2015 |  | B |
| 87 | Latifnejad Roudsari et al 2014 | | English | pregnant women (n=12) and postpartum women(n=10), midwives(n=7), gynecologists(n=7), and non-pregnant women(n=9). | T3, postpartum, and NA | Qualitative | Tonekabon | Not reported | Health facility based | not reported | observations and semi- structured interviews | 2014 |  | B |
| 88 | Abbaspour et al 2014 | | English | Pregnant women (n=4) or postpartum women(n=14) | Not reported | Qualitative | Ahwaz | Urban | Health facility based | 5 | Unstructured interviews | 2013 |  | B |
| 89 | Jamshidimanesh et al 2011 | | English | pregnant women with tendency to elective CS(n=26) | T3 | Qualitative | Tehran | Urban | Health facility based | 3 | in-depth interview | 2011 |  | B |
| 90 | Shirzad et al 2019 | | English | women of varying experiences regarding childbirth(26) | Not reported | Qualitative | Tehran | Urban | Health facility based | 3 | in-depth face-to-face interviews | 2018 |  | A |
| 91 | Abbaspoor et al 2016 | | English | women (4  pregnant, 14 postnatal) | Not reported | Qualitative | Ahvaz | Urban | Health facility based | 5 | in-depth interview | 2012 |  | B |

A - No or few flaws. The study credibility, transferability, dependability, and confirmability is high; B – Some flaws, unlikely to affect the credibility, transferability, dependability, and/or confirmability of the study; C – Some flaws which may affect the credibility, transferability, dependability, and/or confirmability of the study; D – Significant flaws which are very likely to affect the credibility, transferability, dependability, and/or confirmability of the study.institutional review board (IRB)

T1=1st or 2nd trimester,T2=3rd trimester on immediate before labour
