# Supplementary figures and images for: Prevalence of and reasons for women’s, family members’, and health professionals’ preferences for cesarean section in Iran: a mixed-methods systematic review
Source: Reprod Health. 2021 Jan 2;18:3. doi: 10.1186/s12978-020-01047-x (PMC7778821; doi:10.1186/s12978-020-01047-x)

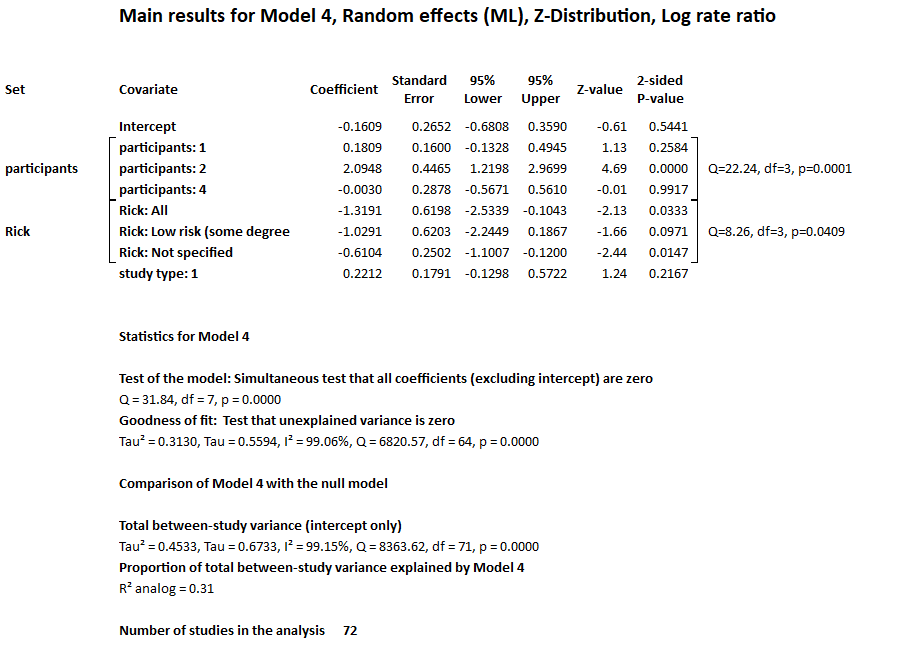

Supplement: Supplementary file 6 — Additional file 6: Meta Regression Result. [file 12978_2020_1047_MOESM6_ESM.docx]
