# Supplementary material for: Prevalence of and reasons for women’s, family members’, and health professionals’ preferences for cesarean section in Iran: a mixed-methods systematic review
Source: Reprod Health. 2021 Jan 2;18:3. doi: 10.1186/s12978-020-01047-x (PMC7778821; doi:10.1186/s12978-020-01047-x)
Supplement: Supplementary file 7 — Additional file 7: Meta Regression Result. [file 12978_2020_1047_MOESM7_ESM.docx]

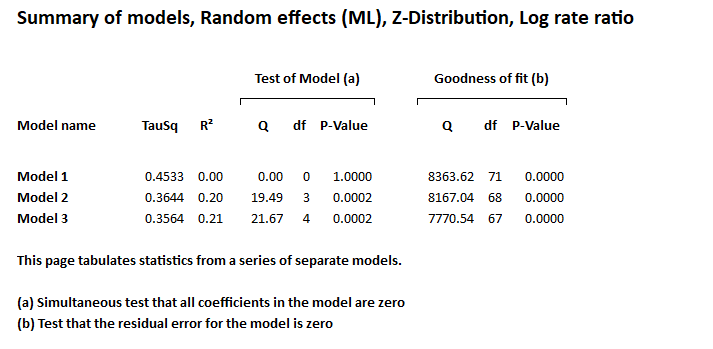


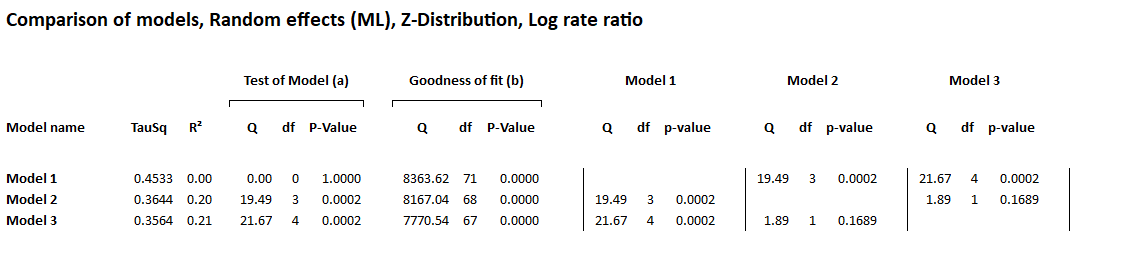


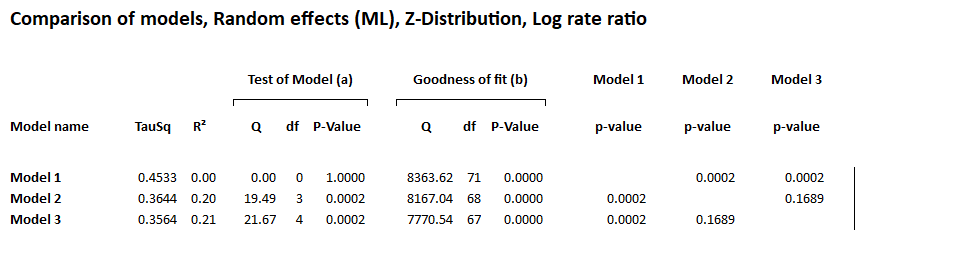


Model 1: Contains participants only

Model 2: Contains participants + Risk

Model 3 contains participants + Risk +time point
