# Supplementary material for: Prevalence of and reasons for women’s, family members’, and health professionals’ preferences for cesarean section in Iran: a mixed-methods systematic review
Source: Reprod Health. 2021 Jan 2;18:3. doi: 10.1186/s12978-020-01047-x (PMC7778821; doi:10.1186/s12978-020-01047-x)
Supplement: Supplementary file 8 — Additional file 8: Reasons for preference for caesarean section reported by women, quantitative studies. [file 12978_2020_1047_MOESM8_ESM.docx]

**S6 Table Reasons for preference for caesarean section reported by women, quantitative studies**

| **Reasons for preference** | **Preference for current pregnancy or index birth** | | | | | | | | | | | | | | |
| --- | --- | --- | --- | --- | --- | --- | --- | --- | --- | --- | --- | --- | --- | --- | --- |
|  | **Payman-2010** | **Andaroon-2017** | **Moradan-2004** | **Norizadeh-2009** | **Bani-2012** | **Bani-2012** | **Negahban-2006** | **shahbazazdegan-2010** | **Aram-2002** | **Vafaee-2013** | **Shakeri_2012** | **Rasoli2019** | **Najafi- Sharjabad2017** | **Siabani2019** | **Masoumi2016** |
|  | **(Mashhad, Pall n=390)** | **(Mashhad, P0, n=220)** | **(Semnan, Pall, n=400)** | **(Marand, Pall, n=450)** | **(Tabriz, Doctors n=153)** | **(Tabriz, Midwifes, n=153)** | **(Rafsanjan, Pall,n=256)** | **(Ardebil, Pall, n=245)** | **(Esfahan,; Pall,n=500)** | **(Shiraz, Partners, n=417)** | **(Zanjan ; P0 n=397)** | **Nulliparous pregnant women(n=211)** | **pregnant women(n=462)** | **pregnant women(n=410)** | **pregnant women(n=150)** |
| **Pain-related fear** | | | | | | | | | | | | | | | |
| Fear of labour pain | 59% |  | 59% | 47.6% | 47.3% | 77% | 52.2% | 43.3% | 37.2% |  | 43.3% |  | 39.5% | 61.46% | NR |
| Afraid of unable to endure (labour) pain |  | 47.2% |  |  |  |  |  |  |  |  |  |  |  |  |  |
| CS is safe and reliable |  |  |  |  | 34.5% |  |  |  |  |  |  |  |  |  |  |
| Safer for mother by CS |  |  |  |  |  |  | 3.8% |  |  |  |  |  |  |  |  |
| To reduce the damage of the pelvic floor |  |  |  | 2% |  |  |  |  |  |  |  |  |  | 41.22% |  |
| Better keeping body image by CS | 2.7% |  |  |  |  |  |  |  | 6.5% |  |  |  |  |  |  |
| Fear of vaginal damages |  |  |  |  |  | 57.5% | 8.8% |  |  | 34.8% |  |  |  | 58.78% | 64.67% |
| Fear of urinary incontinence |  |  |  |  |  | 57.6% |  |  |  |  |  |  |  | 58.78% |  |
| **Fear of VD -perceived risks for the baby** | | | | | | | | | | | | | | | |
| Fear of risk for baby | 12.8% |  |  |  | 70.9% |  | 6.2% |  | 11.5% |  | 33.4% |  |  | NR | 75.33% |
| Perceived that baby would be more clever and less cost |  |  |  | 0.2% |  |  |  |  |  |  |  |  |  | 55.61% |  |
| Healthier baby by CS |  |  |  |  |  |  |  |  |  | 29.3% |  |  |  | 40.97% |  |
| Health of the newborn |  | 19.5% |  |  |  |  |  |  |  |  |  |  |  |  |  |
| Birth trauma to the newborn |  |  |  |  | 67.2% | 71.4% |  |  |  |  |  |  |  |  |  |
| Respiratory trauma to the newborn |  |  |  |  |  |  |  |  |  |  |  |  |  |  |  |
| Fear of low problem |  |  |  |  | 68.6% |  |  |  |  |  |  |  |  |  |  |
| **Cultural and societal related beliefs** | | | | | | | | | | | | | | | |
| Reasonable for schedule and able to select “lucky date” for the birth |  |  |  | 2.6% |  |  |  |  |  |  |  |  |  |  |  |
| **Medical and other reasons** | | | | | | | | | | | | | | | |
| Mistrust to stuff |  | 12.2% |  |  |  |  |  |  |  |  |  |  |  |  |  |
| Maternal health |  | 4.1% |  |  |  |  |  |  |  |  |  |  |  |  |  |
| Doctors/midwives advice | 36.2% |  |  | 18.7% |  |  |  | 17.4% | 35.8% |  |  |  |  |  |  |
| Prior CS |  |  |  | 18.2% |  |  |  | 19.1% |  |  |  |  | 28.5% |  |  |
| Prior Abortion / infertility |  |  | 5.3% |  |  |  |  |  |  |  |  |  |  |  |  |
| Prior negative experience from VD | 6% |  | 3.9% |  |  |  |  |  |  |  |  |  |  |  |  |
| Bad story about VD (by family, friend) | 9.4% | 17.1% |  |  |  |  |  |  |  |  |  |  |  |  |  |
| Fear of environment | 2.7% |  |  |  |  |  |  |  |  |  |  |  |  |  |  |
| Significant others | 2.7% |  |  |  |  |  |  |  |  |  |  |  |  |  |  |
| Doctors /midwife advice |  |  |  |  |  |  |  |  |  |  |  |  | 32% |  |  |
| Spouse/relative advice |  |  |  | 2.3% |  |  | 5% |  | 4% |  |  | 39.3% |  |  |  |
| Tube ligation |  |  | 7% | 6.7% |  |  | 17.5% |  |  |  |  |  |  |  |  |
| Anxiety and psychological pressures |  |  |  |  |  |  |  |  |  |  | 14.7% |  |  |  |  |
| Tube ligation |  |  |  |  |  |  |  |  |  |  | 1.6% |  |  | 41.95% |  |
| Unknown factors |  |  | 18% |  |  |  |  |  |  |  | 7% |  |  |  |  |
