# Supplementary material for: Prevalence of and reasons for women’s, family members’, and health professionals’ preferences for cesarean section in Iran: a mixed-methods systematic review
Source: Reprod Health. 2021 Jan 2;18:3. doi: 10.1186/s12978-020-01047-x (PMC7778821; doi:10.1186/s12978-020-01047-x)
Supplement: Supplementary file 9 — Additional file 9: Qualitative assessment. [file 12978_2020_1047_MOESM9_ESM.docx]

| **Author and date** | **Clear aims appropriate to question** | **Literature review thorough and appropriate** | **Theoretical perspective and design clear and appropriate** | **Sampling strategy explained and appropriate** | **Data collection described and justified** | **Analysis adequately described** | **Findings reflect data** | **Study carried out ethically** | **Researcher reflexivity demonstrated** | **Transferability, relevance and usefulness** | **Quality rating ^1^** |
| --- | --- | --- | --- | --- | --- | --- | --- | --- | --- | --- | --- |
| Ahmad Shirvani et al 2014 | Yes | Good- local context and debate | qualitative research with descriptive- exploratory design | Yes - purposive | In-depth interviews | Yes- good | Yes - plenty of quotes to support themes | IRB was not reported but consents of participants were obtained | No | No | C |
| Javaheri et al 2016 | Yes | Brief but characteristic of Iranian Journal | qualitative research with Phenomenological approach | Yes - purposive | Interviews | Yes- good | Yes - plenty of quotes to support themes | Unclear | No | No | D |
| Rahnama et al 2014 | Yes | Good- local context and debate | Yes justified use of qualitative methodology guided by Theory of Planned Behavior | Yes - purposive | Interviews | Yes- good | Yes - plenty of quotes to support themes | IRB was not reported but consents of participants were obtained | No | No | C |
| Shams et al 2016 | Yes | Good- local context and debate | Yes justified use of qualitative methodology guided by Social Marketing Assessment And Response Tool | Yes - purposive | Semi-structured interviews and Focus group discussions | Yes - brief description | Yes - plenty of quotes to support themes | Yes | No | No | C |
| Shahavi et al 2014 | Yes | Good- local context and debate | qualitative research with Phenomenological approach | Yes - purposive | Semi-structured nterviews and In-depth interviews | Yes - brief description | Yes - plenty of quotes to support themes | Yes | No | No | C |
| Vaziri et al 2013 | Yes | Good- local context and debate | qualitative reserearch | yes- purposive | Semi-structured nterviews | Yes- good | Yes - plenty of quotes to support themes | Yes | No | yes | B |
| Hajiyan et al 2011 | Yes | Good- local context and debate | Content-analysis , Semi- tructured interviews | yes- purposive | Semi-structured nterviewsused Delphi method and Focus group discussions | yes-brief description | Yes - plenty of quotes to support themes | IRB was not reported but consents of participants were obtained | No | yes | C |
| Borghei et al 2016 | Yes | Good- local context and debate | Content-analysis , Semi- tructured interviews and indepth interviews | yes- purposive | semi- stuctured interviews | yes-good | Yes - plenty of quotes to support themes | No | yes | yes | B |
| Darvishi et al 2012 | Yes | Good- local context and debate | Content-analysis , Semi- tructured interviews | yes- purposive | Semi-structured nterviews | yes-brief description | Yes - plenty of quotes to support themes | IRB was not reported but consents of participants were obtained | No | yes | C |
| Abbaspoor et al 2014 | Yes | Good- local context and debate | Content-analysis , Semi- tructured interviews and indepth interviews | yes- purposive | semi- stuctured interviews and indepth interviews | yes-brief description | Yes - plenty of quotes to support themes | Yes | No | No | C |
| Beyrami et al 2011 | Yes | Good- local context and debate | Content-analysis , Semi- tructured interviews and indepth interviews | yes- purposive | semi- stuctured interviews and indepth interviews | yes-brief description | Yes - plenty of quotes to support themes | Yes | No | No | C |
| Vedadhir et al 2012 | Yes | Good- local context and debate | qualitative research | yes- purposive | Interviews | Yes- good | No | IRB and consents of participants were not reported | No | Yes | C |
| Rahnama et al 2016 | Yes | Good- local context and debate | qualitative research-framed by the Theory of Planned Behavior | yes- purposive | in-depth interviews and focus group | Yes- good | Yes - plenty of quotes to support themes | Yes | No | No | C |
| Mobarakabadi et al 2015 | Yes | Good- local context and debate | qualitative research | yes- purposive | n depth unstructured interviews | Yes- good | Yes - plenty of quotes to support themes | Yes | No | Yes | B |
| Shahoei et al 2014 | Yes | Good- local context and debate | qualitative research | yes- purposive | semi structured nterviews | Yes- good | Yes - plenty of quotes to support themes | Yes | No | Yes | B |
| Faisal et al 2014 | Yes | Good- local context and debate | qualitative research | yes- purposive | in-depth semi - structured interviews | Yes- good | Yes - plenty of quotes to support themes | Yes | No | Yes | B |
| Hajian et al 2013 | Yes | Good- local context and debate | mixed methods study | yes- purposive | focus group discussions | Yes- good | Yes - plenty of quotes to support themes | IRB and consents of participants were not reported | No | Yes | B |
| Bagheri et al 2013 | Yes | Good- local context and debate | qualitative research | yes- purposive | semi-structured interviews | Yes- good | Yes - plenty of quotes to support themes | Yes | No | Yes | B |
| Sanavi et al 2012 | Yes | Brief | qualitative research | not reported | focus group discussions | yes-brief description | yes-Few plenty of quotes to support themes | No | No | No | D |
| Yazdizadeh et al 2011 | Yes | Good- local context and debate | qualitative research | yes- purposive | in-depth interviews and document analyses | Yes- good | Yes - plenty of quotes to support themes | IRB not reported- consents of participants wereobtained | yes | Yes | A |
| Latifnejad Roudsari et al 2015 | Yes | Good- local context and debate | qualitative research -Focused ethnography | yes- purposive | Semi-structured nterviews and articipant observations | Yes- good | Yes - plenty of quotes to support themes | Yes | No | Yes | B |
| Latifnejad-Roudsari et al 2014 | Yes | Good- local context and debate | Focused ethnographic method | yes- purposive | observations and semi- structured interviews | Yes- good | Yes - plenty of quotes to support themes | Yes | No | Yes | B |
| Abbaspoor et al 2013 | Yes | Good- local context and debate | qualitative research | yes- purposive | Unstructured interviews | Yes- good | Yes - plenty of quotes to support themes | Yes | No | Yes | B |
| Jamshidimanesh et al 2011 | yes | Good- local context and debate | qualitative research | yes- purposive | in-depth interview | Yes- good | Yes - plenty of quotes to support themes | IRB and consents of participants were not reported | No | Yes | B |
| Shirzad et al 2019 | yes | Good- local context and debate | Content-analysis , Semi- tructured interviews | yes- purposive | in-depth face-to-face interviews | Yes- good | Yes - plenty of quotes to support themes | Yes | Yes | Yes | A |
| Abbaspoor et al 2016 | yes | Good- local context and debate | Content-analysis , Semi- tructured interviews | yes- purposive | in-depth face-to-face interviews | yes-brief description | Yes - plenty of quotes to support themes | Yes | no | Yes | B |

A No or few flaws. The study credibility, transferability, dependability, and confirmability is high; B – Some flaws, unlikely to affect the credibility, transferability, dependability, and/or confirmability of the study; C – Some flaws which may affect the credibility, transferability, dependability, and/or confirmability of the study; D – Significant flaws which are very likely to affect the credibility, transferability, dependability, and/or confirmability of the study. institutional review board (IRB)
