# Supplementary material for: Prevalence of and reasons for women’s, family members’, and health professionals’ preferences for cesarean section in Iran: a mixed-methods systematic review
Source: Reprod Health. 2021 Jan 2;18:3. doi: 10.1186/s12978-020-01047-x (PMC7778821; doi:10.1186/s12978-020-01047-x)
Supplement: Supplementary file 10 — Additional file 10: Tables 4–29 theCERQual findings. [file 12978_2020_1047_MOESM10_ESM.docx]

| Finding#1 | |
| --- | --- |
| **Deep rooted fear of labour pain and vaginal birth** | |
| **Assessment for each CERQual component** | |
| Methodological limitation | Minor concerns because 18 studies did not report reflexivity; 9 studies did not report ethical consideration; one study did not report sampling strategy; 5 studies did not report data analysis completely. However , these may not have influenced the finding. |
| Coherence | Minor concerns because five studies some women had controversial feelings towards birth pain |
| Relevance | Minor concerns because most data were from Nulliparous Pregnant women |
| Adequacy | No or very minor concerns |
| **Overall CERQual assessment** | |
| **Moderate confidence** | Due to minor concerns about methodological limitation, coherence, and relevance |
| **Contributing studies** | |
| Rahnama 2015  Sanavi 2012  Ahmad Shirvani 2014  Rahnama 2016  Shams 2016  Vaziri 2013  Borghei-2016  Darvishi 2012  Bayrami 2011  Mobarakabadi 2015  Javaheri 2016  Hajian 2013  Jamshidimanesh 2011  Bagheri 2013  Vedadhir 2011  Shahoei 2014  Abbaspour 2014  Latifnejad Roudsari 2014  Faisal 2014 | |

| Finding # 2 | |
| --- | --- |
| **Irreversible damage to body and sexual function** | |
| **Assessment for each CERQual component** | |
| Methodological limitation | Minor concerns because12studies did not report reflexivity; 5 studies did report ethical concideration;4 studies did report data analysis brief. However , these may not have influenced the finding. |
| Coherence | No or very minor concerns |
| Relevance | Moderate concerns because all studies setting is urban. |
| Adequacy | No or very minor concerns |
| **Overall CERQual assessment** | |
| **Moderate confidence** | Due to minor concerns about methodological limitations; No or very minor concerns about coherence and adequacy; and moderate concern about relevance |
| **Contributing studies** | |
| Rahnama 2016  Yazdizadeh 2011  Rahnama 2015  Shams 2016  Javaheri 2016  Hajian 2013  Hajiyan 2011  Jamshidimanesh 2011  Vedadhir 2011  Abbaspour 2014  Latifnejad Roudsari 2014  Faisal 2014  Abbaspoor2016 | |

| Finding # 3 | |
| --- | --- |
| **Safety (mother/ baby) and comfort** | |
| **Assessment for each CERQual component** | |
| Methodological limitation | Minor concerns because 16 studies did not report reflexivity; 10 studies did report ethical concideration;4 studies did report data analysis briefly. However , these may not have influenced the finding. |
| Coherence | Minor concerns because 4 studies women believed that safety of fetus was guaranteed during NVD. |
| Relevance | No or very minor concerns |
| Adequacy | No or very minor concerns |
| **Overall CERQual assessment** | |
| **Moderate confidence** | Due to minor concerns about methodological limitations and coherence ;No or very minor concerns about relevance and adequacy. |
| **Contributing studies** | |
| Rahnama 2015  Ahmad Shirvani 2014  Rahnama 2016  Shams 2016  Yazdizadeh 2011  Borghei-2016  Bayrami 2011  Mobarakabadi 2015  Hajian 2013  Jamshidimanesh 2011  Hajiyan 2011  Vedadhir 2011  Shahoei. 2014  Shahoei 2014  Abbaspour 2014  Abbaspour 2014  Latifnejad Roudsari 2015  Faisal 2014  Sanavi 2012 | |

| Finding # 4 | |
| --- | --- |
| **Social convenience of birthing to time (time scheduling)** | |
| **Assessment for each CERQual component** | |
| Methodological limitation | Minor concerns because all studies did not report reflexivity; 2 studies did report ethical concideration;2 studies did report data analysis briefly. However , these may not have influenced the finding. |
| Coherence | No or very minor concerns. |
| Relevance | Moderate concerns because most studies(4) setting is urban and 2 studies did not reported setting of study. |
| Adequacy | Moderate concerns due to few studies reported this finding. |
| **Overall CERQual assessment** | |
| **Low confidence** | Due to minor concerns about methodological limitations, and moderate concerns about relevance and adequacy. |
| **Contributing studies** | |
| Rahnama 2015  Sanavi 2012  Rahnama 2016  Darvishi 2012  Mobarakabadi 2015  Javaheri 2016  Abbaspour 2014  Jamshidimanesh 2011  Vedadhir 2011  Ahmad Shirvani 2014 | |

| Finding # 5 | |
| --- | --- |
| **Religious beliefs** | |
| **Assessment for each CERQual component** | |
| Methodological limitation | Moderate concerns because all studies did not report reflexivity; 5 studies did report ethical concideration;2 studies did report data analysis briefly. However , these may not have influenced the finding. |
| Coherence | No or very minor concerns. |
| Relevance | No or very minor concerns. |
| Adequacy | No or very minor concerns. |
| **Overall CERQual assessment** | |
| **Moderate confidence** | Due to moderate concerns about methodological limitations ;No or very minor concerns about relevance ,coherence, and adequacy |
| **Contributing studies** | |
| Ahmad Shirvani 2014  Rahnama 2016  Bayrami 2011  Abbaspour 2014  Mobarakabadi 2015  Javaheri 2016  Hajian 2013  Hajiyan 2011  Latifnejad Roudsari 2015 | |

| Finding # 6 | |
| --- | --- |
| **Cultural beliefs (having role models; modernity, capability to do vaginal birth)** | |
| **Assessment for each CERQual component** | |
| Methodological limitation | Minor concerns because one study did report reflexivity; 4 studies did report ethical consideration; one study did report data analysis briefly. However , these may not have influenced the finding. |
| Coherence | No or very minor concerns. |
| Relevance | Moderate concerns because most studies(6) setting is urban and 3 studies did not reported setting of study |
| Adequacy | No or very minor concerns |
| **Overall CERQual assessment** | |
| **Moderate confidence** | Due to minor concerns about methodological limitations ;No or very minor concerns about adequacy and coherence; Moderate concerns about Relevance. |
| **Contributing studies** | |
| Latifnejad Roudsari 2015  Abbaspour 2014  Yazdizadeh 2011  Mobarakabadi 2015  Hajiyan 2011  Hajian 2013  Bagheri 2013  Vedadhir 2011  Latifnejad Roudsari 2014 | |

| Finding # 7 | |
| --- | --- |
| **Influence of information about birth from family, friends, and doctors** | |
| **Assessment for each CERQual component** | |
| Methodological limitation | Moderate concerns because one study did report reflexivity;11 studies did report ethical concideration;5 study did report data analysis briefly. However , these may not have influenced the finding. |
| Coherence | Minor concerns because in most studies, information and experiences with others are encouraged women to choose CS, but one study has addressed the role of midwives in providing information to women for choosing NVD. |
| Relevance | Moderate concerns because most studies setting is urban. |
| Adequacy | No or very minor concerns. |
| **Overall CERQual assessment** | |
| **Moderate confidence** | Due to moderate concerns about methodological limitations and Relevance; minor concerns about coherence; No or very minor concerns about adequacy. |
| **Contributing studies** | |
| Latifnejad Roudsari 2014  Shahoei 2014  Abbaspour 2014  Ahmad Shirvani 2014  Sanavi 2012  Yazdizadeh 2011  Borghei-2016  Shams 2016  Rahnama 2016  Abbaspour 2014  Shahoei 2014  Vaziri 2013  Mobarakabadi 2015  Javaheri 2016  Hajiyan 2011  Hajian 2013  Jamshidimanesh 2011  Bagheri 2013  Faisal 2014  Vedadhir 2011  Darvishi 2012 | |

| Finding # 8 | |
| --- | --- |
| **Women’s previous birth experience** | |
| **Assessment for each CERQual component** | |
| Methodological limitation | Minor concerns because all studies did not report reflexivity; 2 studies did report ethical concideration;3 studies did report data analysis briefly. However , these may not have influenced the finding. |
| Coherence | Moderate concerns because in eight studies, previous experience has led to an increase in women's desire for cesarean section, while in two studies, it has increased the likelihood of natural delivery as a result of previous experience. In one study, despite the previous unpleasant experience of normal delivery and fear of it, normal delivery was preferred to cesarean section. |
| Relevance | No or very minor concerns |
| Adequacy | No or very minor concerns |
| **Overall CERQual assessment** | |
| **Moderate confidence** | Due to minor concerns about methodological limitations ;moderate concerns about coherence; No or very minor concerns about adequacy and relevance |
| **Contributing studies** | |
| Vaziri 2013  Ahmad Shirvani 2014  Latifnejad Roudsari 2014  Bayrami 2011  Shahoei 2014  Jamshidimanesh 2011  Bagheri 2013  Abbaspour 2014  Rahnama 2016  Shahoei 2014 | |

| Finding # 9 | |
| --- | --- |
| **Women’s preferences informed by availability (i.e. what they or insurance can pay)** | |
| **Assessment for each CERQual component** | |
| Methodological limitation | Minor concerns because all studies did not report reflexivity; one study did not report ethical consideration; one study did report data analysis briefly. However , these may not have influenced the finding. |
| Coherence | No or very minor concerns. |
| Relevance | Moderate concerns because two studies setting is urban and one of them is mixed. |
| Adequacy | Moderate concerns due to few studies reported this finding |
| **Overall CERQual assessment** | |
| **Moderate confidence** | Due to minor concerns about methodological limitations ;moderate concerns about adequacy and relevance ;No or very minor concerns about coherence. |
| **Contributing studies** | |
| Mobarakabadi 2015  Ahmad Shirvani 2014  Shams 2016 | |

| Finding # 10 | |
| --- | --- |
| **CS is now safe/r option for birth** | |
| **Assessment for each CERQual component** | |
| Methodological limitation | Minor concern because 2 studies brief description about analysis, 2 studies did not report ethical consideration, and 3 studies did not report reflexivity |
| Coherence | No or minor concern. |
| Relevance | Minor concern; 3 studies carried out in urban and 2 studies did not report the setting of study |
| Adequacy | Minor concern due to few studies reported this finding. Even though the studies had richness. |
| **Overall CERQual assessment** | |
| **Moderate confidence** | Due to minor concerns about methodological limitation and adequacy. |
| **Contributing studies** | |
| Latifnejad Roudsari 2014  Darvishi 2012  Hajiyan 2011  Bagheri 2013  Yazdizadeh 2011 | |

| Finding # 11 | |
| --- | --- |
| **Convenience of birthing to time (work scheduling)** | |
| **Assessment for each CERQual component** | |
| Methodological limitation | Moderate concern because 4 studies did not report reflexivity, 3 studies did not have transferability, relevance and usefulness, 1 study analysis did not adequately describe, 1 study did not report ethical consideration. |
| Coherence | No or minor concern. |
| Relevance | Minor concern; 2 studies carried out in urban, 2 studies carried out in urban and rural setting, 1 study did not report the setting of study. |
| Adequacy | Modarete concern due to few studies reported this finding. |
| **Overall CERQual assessment** | |
| **Modarate confidence** | Due to moderate concern about methodological concern and minor concern about adequacy. |
| **Contributing studies** | |
| Yazdizadeh 2011  Shams 2016  Rahnama 2016  Darvishi 2012  Bagheri 2013 | |

| Finding # 12 | |
| --- | --- |
| **Patient pushes doctor to do CS** | |
| **Assessment for each CERQual component** | |
| Methodological limitation | Minor concern because 2 studies did not report reflexivity, 1 study did not have transferability, relevance and usefulness, 1 study did report data analysis briefly, 1 study did not report ethical consideration; However, these may not have influenced the finding. |
| Coherence | No or minor concern. |
| Relevance | No or minor concern; participants were women and healthcare providers, 1 study participants contributed from urban and rural. 1 study did not report the setting of study, 1 study carried out in urban |
| Adequacy | Moderate concern because few studies supported this finding. |
| **Overall CERQual assessment** | |
| **Moderate confidence** | Due to minor concern about methodological limitation and moderate concern about adequacy. |
| **Contributing studies** | |
| Darvishi 2012  Yazdizadeh 2011  Bagheri 2013 | |

| Finding # 13 | |
| --- | --- |
| **Legal issues** | |
| **Assessment for each CERQual component** | |
| Methodological limitation | Moderate concern because 3 studies did not report reflexivity, 2 studies analysis did not adequately describe, 2 studies did not report ethical consideration |
| Coherence | No or minor concern |
| Relevance | No or minor concern; 1 study carried out in urban and rural setting, 2 studies carried out urban, 1 study did not report setting of the study |
| Adequacy | Moderate concern due to few studies reported this finding |
| **Overall CERQual assessment** | |
| **Moderate confidence** | Due to moderate concerns about methodological limitation and adequacy; No or very minor concerns about relevance and relevance |
| **Contributing studies** | |
| Darvishi 2012  Yazdizadeh 2011  Hajiyan 2011  Faisal 2014 | |

| Finding # 14 | |
| --- | --- |
| **Vaginal delivey fees do not worse the time paid on it** | |
| **Assessment for each CERQual component** | |
| Methodological limitation | Minor concerns because 4 studies did not report reflexivity, 1 study Analysis did not adequately describe, one study did not report ethical consideration; However , these may not have influenced the finding. |
| Coherence | Minor concerns because in one study specialists have controversial opinions regarding the effect of changing the vaginal delivery tariff on the CS rate. |
| Relevance | No or minor concern |
| Adequacy | Moderate concern because few studies supported this finding. |
| **Overall CERQual assessment** | |
| **Moderate confidence** | Due to minor concerns about methodological concern and coherence; Moderate concern about adequacy and No or minor concern about relevance. |
| **Contributing studies** | |
| Darvishi 2012  Hajiyan 2011  Bagheri 2013  Faisal 2014  Yazdizadeh 2011 | |

| Finding # 15 | |
| --- | --- |
| **Lack of respectful, dignified, and supportive communication with women** | |
| **Assessment for each CERQual component** | |
| Methodological limitation | Moderate concerns because14 studies did not report reflexivity, 6 studies did not have transferability, relevance and usefulness, 3 studies Analysis did not adequately described, 3 study did not report ethical consideration and 1 studies did not have Literature review thorough and appropriate and 1 studies did not report findings reflect data |
| Coherence | Minor concerns because women have controversial opinions, some of them had good experience from healthcare providers’ communication and approach and some of them had bad experiences . |
| Relevance | No or minor concern |
| Adequacy | No or minor concern |
| **Overall CERQual assessment** | |
| **Moderate confidence** | Due to moderate concerns about methodological limitation; Minor concerns about coherence; No or minor concerns about relevance and adequacy. |
| **Contributing studies** | |
| Ahmad Shirvani 2014  Shams 2016  Vaziri 2013  Shahoei 2014  Latifnejad Roudsari 2014  Abbaspour 2014  Latifnejad Roudsari 2015  Mobarakabadi 2015  Yazdizadeh 2011  Javaheri 2016  Jamshidimanesh 2011  Faisal 2014  Vedadhir 2011  Bayrami 2011  Bagheri 2013  Shirzad2019 | |

| Finding # 16 | |
| --- | --- |
| **Lack of providing enough information to women** | |
| **Assessment for each CERQual component** | |
| Methodological limitation | Moderate concerns because 5 studies did not report reflexivity, 3 studies did not have transferability, relevance and usefulness, 3 studies Analysis did not adequately described, 4 study did not report ethical consideration, 1 study did not have Literature review thorough and appropriate, 1 study did not or few report findings reflect data, and 1 study did not report sampling strategy explained and appropriate. |
| Coherence | No or minor concerns. |
| Relevance | Minor concerns; 3 studies carried out in urban, 2 studies carried out in urban and rural, 1 studies did not report the setting of the study. Participants were women and health care providers. |
| Adequacy | No or minor concerns |
| **Overall CERQual assessment** | |
| **Moderate confidence** | Due to moderate concerns about methodological limitation; No or minor concerns about coherence and adequacy; Minor concerns about relevance. |
| **Contributing studies** | |
| Sanavi 2012  Yazdizadeh 2011  Shams 2016  Hajiyan 2011  Hajian 2013  Ahmad Shirvani 2014  Shirzad2019 | |

| Finding # 17 | |
| --- | --- |
| **Mistrust** | |
| **Assessment for each CERQual component** | |
| Methodological limitation | Minor concern because 3 studies did not report reflexivity, 1 study did not report ethical consideration, 1 studies did not or few report findings reflect data |
| Coherence | No or minor concern |
| Relevance | Minor concern; most of the studies were urban, one study did not report setting of the study |
| Adequacy | Moderated concerns due to few studies reported this finding |
| **Overall CERQual assessment** | |
| **Moderate confidence** | Due to minor concern about methodological limitation and relevance; moderate concern about adequacy ;No or minor concerns about coherence |
| **Contributing studies** | |
| Mobarakabadi 2015  Faisal 2014  Bagheri2013  Yazdizadeh 2011  Shirzad2019 | |

| Finding #18 | |
| --- | --- |
| **Lack of skilled and experienced doctors/midwives during labor and vaginal birth** | |
| **Assessment for each CERQual component** | |
| Methodological limitation | Minor concern because one study analysis did not adequately describe and did not have reflexivity and transferability. |
| Coherence | No or minor concern |
| Relevance | No or minor concern |
| Adequacy | Moderate concerns, as only 3 studies with relatively thin data reported this finding. |
| **Overall CERQual assessment** | |
| **Moderate confidence** | Due to minor concern about methodological limitation ; moderate concerns about adequacy ;No or minor concerns about coherence and relevance |
| **Contributing studies** | |
| Shams 2016  Yazdizadeh 2011  Shirzad2019 | |

| Finding # 19 | |
| --- | --- |
| **Too little value placed on midwifery care** | |
| **Assessment for each CERQual component** | |
| Methodological limitation | Minor concerns because two studies did not have reflexivity .However , these may not have influenced the finding. |
| Coherence | No or minor concerns |
| Relevance | No or minor concerns |
| Adequacy | Modarate concerns due to few studies reported this finding |
| **Overall CERQual assessment** | |
| **Moderate confidence** | Due to minor concerns about methodological limitation and adequacy ; No or minor concerns about coherence and relevance |
| **Contributing studies** | |
| Yazdizadeh 2011  Mobarakabadi 2015  Bagheri 2013  Shirzad2019 | |

| Finding # 20 | |
| --- | --- |
| **Financial and legal conflicts** | |
| **Assessment for each CERQual component** | |
| Methodological limitation | No or minor concern |
| Coherence | No or minor concern |
| Relevance | No or minor concern |
| Adequacy | Serious concern because just one study report this finding |
| **Overall CERQual assessment** | |
| **very low confidence** | Due to serious concern about adequacy, |
| **Contributing studies** | |
| Yazdizadeh 2011 | |

| Finding # 21 | |
| --- | --- |
| **Physical condition of birth facility (comfortable, calming, clean birth environment)** | |
| **Assessment for each CERQual component** | |
| Methodological limitation | Moderate concerns because 10 studies did not report reflexivity,3 studies did not have transferability, relevance and usefulness, 3 studies Analysis did not adequately described, one study did not reflect Findings data, 2 study did not report ethical consideration |
| Coherence | No or minor concern |
| Relevance | No or minor concern |
| Adequacy | No or minor concern |
| **Overall CERQual assessment** | |
| **Moderate confidence** | Due to moderate concerns about methodological limitation, No or minor concerns about coherence, adequacy , and relevance |
| **Contributing studies** | |
| Rahnama 2016  Vaziri 2013  Bayrami 2011  Mobarakabadi 2015  Hajiyan 2011  Vedadhir 2011  Shams 2016  Latifnejad Roudsari 2014  Abbaspour 2014  Yazdizadeh 2011  Bagheri 2013  Shirzad2019 | |

| Finding #22 | |
| --- | --- |
| **Physical examination and procedures (asking permission, privacy, painful vaginal examination, unnecessary vaginal examinations/interventions)** | |
| **Assessment for each CERQual component** | |
| Methodological limitation | Moderate concerns because 9 studies did not report reflexivity,5 studies did not have transferability, relevance and usefulness, 4 studies analysis did not adequately described, 2 study did not reflect findings data, 3 study did not report ethical consideration, one study did not report sampling strategy, one study did not report literature review thorough and appropriate |
| Coherence | No or minor concern |
| Relevance | No or minor concern |
| Adequacy | No or minor concern |
| **Overall CERQual assessment** | |
| **Moderate confidence** | Due to moderate concerns about methodological limitation; No or minor concerns about adequacy; No or minor concerns about coherence and relevance |
| **Contributing studies** | |
| Yazdizadeh 2011  Abbaspour 2014  Sanavi 2012  Latifnejad Roudsari 2014  Rahnama 2016  Hajiyan 2011  Jamshidimanesh 2011  Vedadhir 2011  Mobarakabadi 2015  Bayrami 2011  Shirzad2019 | |

| Finding # 23 | |
| --- | --- |
| **Continuous and organized care (lack of neglect and abandonment, timely care)** | |
| **Assessment for each CERQual component** | |
| Methodological limitation | Minor concerns because 5 studies did not report reflexivity,2 studies did not have transferability, relevance and usefulness,2 studies analysis did not adequately described,3 study did not report ethical consideration. |
| Coherence | No or minor concerns |
| Relevance | No or minor concerns |
| Adequacy | minor concerns because in three studies with relatively thin data reported this finding. |
| **Overall CERQual assessment** | |
| **Moderate confidence** | Due to minor concerns about methodological limitation; Moderate concerns about adequacy; No or minor concerns about relevance |
| **Contributing studies** | |
| Hajiyan 2011  Hajian 2013  Bagheri 2013  Jamshidimanesh 2011  Yazdizadeh 2011  Shams 2016  Shirzad2019 | |

| Finding # 24 | |
| --- | --- |
| **Limited availability of pain relief procedures** | |
| **Assessment for each CERQual component** | |
| Methodological limitation | Moderate concerns because 6 studies did not report reflexivity, 5 studies did not have transferability, relevance and usefulness,4 studies analysis did not adequately described, 1 study did not report ethical consideration, 1 study did not report literature review thorough and appropriate |
| Coherence | No or minor concern |
| Relevance | Minor concerns; most of the studies carried out in urban setting 1 studies carried out in urban and rural setting |
| Adequacy | Minor concern because in two studies with relatively thin data reported this finding. |
| **Overall CERQual assessment** | |
| **Moderate confidence** | Due to moderate concern about methodological limitation and minor concern about adequacy and relevance; No or minor concerns about coherence |
| **Contributing studies** | |
| Ahmad Shirvani 2014  Shams 2016  Abbaspour 2014  Bayrami 2011  Javaheri 2016  Bagheri 2013  Shirzad2019 | |

| Finding # 25 | |
| --- | --- |
| **Lack of partner/family companion during labour/delivery** | |
| **Assessment for each CERQual component** | |
| Methodological limitation | Minor concerns because 4 studies did not report reflexivity,2 studies did not have transferability, relevance and usefulness, 1 studies Analysis did not adequately described, 1 study did not report ethical consideration, 1 study did not report Literature review thorough and appropriate |
| Coherence | No or minor concern |
| Relevance | Minor concern ; 4 studies carried out in urban setting, 1 study did not report the setting of study |
| Adequacy | No or minor concern |
| **Overall CERQual assessment** | |
| **Moderate confidence** | Due to minor concern about methodological limitation and relevance; No or minor concerns about coherence and relevance |
| **Contributing studies** | |
| Abbaspour 2014  Yazdizadeh 2011  Javaheri 2016  Bagheri 2013  Faisal 2014  Shirzad2019 | |

| Finding # 26 | |
| --- | --- |
| **Lack of practical birth guidelines and collaborative midwife-obstetrician models of care** | |
| **Assessment for each CERQual component** | |
| Methodological limitation | No or minor concern |
| Coherence | No or minor concern |
| Relevance | No or minor concern |
| Adequacy | Serious concerns because just two studies supported this finding. |
| **Overall CERQual assessment** | |
| **very low confidence** | Due to serious concerns about adequacy. |
| **Contributing studies** | |
| Yazdizadeh 2011  Shirzad2019 | |
