# Supplementary material for: Prevalence of and reasons for women’s, family members’, and health professionals’ preferences for cesarean section in Iran: a mixed-methods systematic review
Source: Reprod Health. 2021 Jan 2;18:3. doi: 10.1186/s12978-020-01047-x (PMC7778821; doi:10.1186/s12978-020-01047-x)
Supplement: Supplementary file 11 — Additional file 11: Summaries of initial concepts, emergent themes, final themes, and supporting quotes. [file 12978_2020_1047_MOESM11_ESM.docx]

| Women’s factors | Women’s beliefs | Deep rooted fear of labour pain and vaginal birth |
| --- | --- | --- |
|  |  | Irreversible damage to body appearance and sexual function |
|  |  | Safety (mother/baby) and comfort |
|  |  | Social convenience of birthing to time (time scheduling) |
|  |  | Religious beliefs |
|  |  | Cultural beliefs (having role models; modernity, capability to do vaginal birth) |
|  | Women’s experiences | Influence of information about birth from friends, family, colleagues, doctor |
|  |  | Women’s previous birth experience |
|  | Women’s resources | Women’s preferences informed by availability (i.e. what they or insurance can pay) |
| Healthcare professional factors | HCPs’ beliefs | Belief CS is now safe/r option for birth |
|  |  | Convenience of birthing to time (work scheduling) |
|  |  | Patient pushes doctor to do CS |
|  |  | Legal issues |
|  | Financial drivers, financial means and burdens | Financial income more important than health outcomes |
|  | Communication between women and HCPs | Lack of respectful, dignified, and supportive communication with women |
|  |  | Lack of providing enough information to women |
|  |  | Mistrust |
|  | HCP’s training, skills, experience, competence, accessibility, number, motivation, and influence | Lack of skills and experience during labor and vaginal birth |
|  | Communication between doctors and midwives | Too little value placed on midwifery care |
|  |  | Financial conflicts |
| Health organization, facility, or system factors | Standards of care in birth facilities | Physical condition of birth facility (comfortable, calming, clean birth environment) |
|  |  | Physical examination and unnecessary procedures (asking permission, painful vaginal examination, unnecessary vaginal examination) |
|  |  | Continuous and organized care (lack of neglect and abandonment, timely care) |
|  |  | Lack of privacy |
|  | Birth policies | Limited availability of pain relief procedures |
|  |  | Lack of partner/family companion during labour/delivery |
|  |  | Lack of practical birth guidelines and collaborative midwife-obstetrician models of care |
